# Molecularly Imprinted Polymer-Based Sensors for Priority Pollutants

**DOI:** 10.3390/s21072406

**Published:** 2021-03-31

**Authors:** Mashaalah Zarejousheghani, Parvaneh Rahimi, Helko Borsdorf, Stefan Zimmermann, Yvonne Joseph

**Affiliations:** 1 Institute of Electronic and Sensor Materials, Faculty of Materials Science and Materials Technology, Technische Universität Bergakademie Freiberg, 09599 Freiberg, Germany; Parvaneh.Rahimi@esm.tu-freiberg.de (P.R.); Yvonne.Joseph@esm.tu-freiberg.de (Y.J.); 2 Department Monitoring and Exploration Technologies, Helmholtz Centre for Environmental Research-UFZ, 04318 Leipzig, Germany; helko.borsdorf@ufz.de; 3 Department of Sensors and Measurement Technology, Institute of Electrical Engineering and Measurement Technology, Leibniz University Hannover, 30167 Hannover, Germany; zimmermann@geml.uni-hannover.de

**Keywords:** molecularly imprinted polymer, priority pollutant, U.S. Environmental Protection Agency, sensor, disposable sensor, optical sensor, electrochemical sensor, chemiresistor, quartz crystal microbalance, quartz crystal tuning fork

## Abstract

Globally, there is growing concern about the health risks of water and air pollution. The U.S. Environmental Protection Agency (EPA) has developed a list of priority pollutants containing 129 different chemical compounds. All of these chemicals are of significant interest due to their serious health and safety issues. Permanent exposure to some concentrations of these chemicals can cause severe and irrecoverable health effects, which can be easily prevented by their early identification. Molecularly imprinted polymers (MIPs) offer great potential for selective adsorption of chemicals from water and air samples. These selective artificial bio(mimetic) receptors are promising candidates for modification of sensors, especially disposable sensors, due to their low-cost, long-term stability, ease of engineering, simplicity of production and their applicability for a wide range of targets. Herein, innovative strategies used to develop MIP-based sensors for EPA priority pollutants will be reviewed.

## 1. Introduction

The U.S. EPA Priority Pollutant List was developed in 1977 from the Toxic Pollutant List to categorize individual pollutants more specifically for practical and regulatory monitoring of water quality [1]. In addition to this list, EPA has also provided a list of hazardous air pollutants containing 187 chemicals [2]. In this review, we focus on the EPA Priority Pollutant List, as molecularly imprinted polymers (MIPs) have been mostly used for the selective recognition of target molecules in liquid samples (Table S1). EPA priority pollutants include different important groups of chemicals including pesticides, polycyclic aromatic hydrocarbons (PAHs), polychlorinated biphenyls (PCBs), phthalate esters, heavy metals, halogenated compounds, BTEX compounds (benzene, toluene, ethylbenzene and xylene), phenol and nitrophenol compounds. All of these chemicals cover a broad range of organic and inorganic chemicals with various physical-chemical properties. Unlike acutely toxic chemicals, most of the listed EPA Priority Pollutants have chronic toxicity, which means long-term exposure to some concentrations of these chemicals can cause severe and irrecoverable health effects. Due to the importance of EPA priority pollutants, many studies have been conducted to develop efficient techniques and platforms for detection, quantification, and removal of these chemicals. Among them, MIPs have attracted considerable research interest as selective sorbent materials and biomimetic recognition elements for environmental applications.

**Figure 1:**
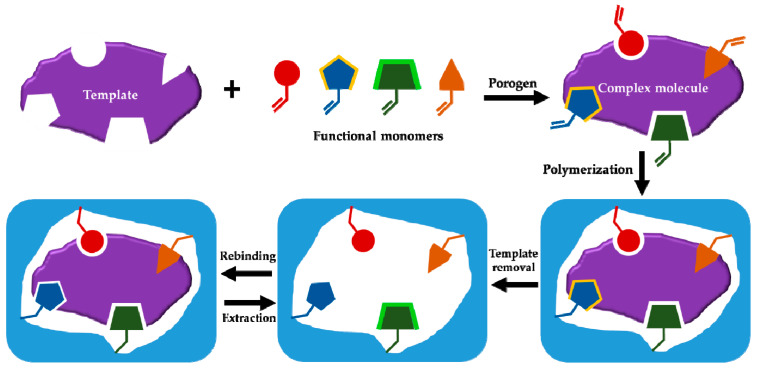
Schematic representation of MIP preparation.

MIPs or plastic antibodies are highly cross-linked polymers in which tailor-made recognition sites are imprinted for specific target molecules known as the template molecules [3,4]. The imprinting process is based on a preliminary complexation of the template molecule and selected functional monomers in a suitable solvent (porogen) via different kinds of covalent, non-covalent and semi-covalent interactions. After complexation, the monomers are fixed in their position around the template molecule by the free radical polymerization method or the controlled/living radical polymerization techniques [5]. After removal of the template molecule, the imprinted recognition site is remained within the highly cross-linked polymer matrix and have a complementary size, shape, and spatial position of the functional groups towards the template (Figure 1). The first positive results for MIPs were obtained in early 1970s using covalent approach. In this method, the complexation between template and functional monomers are based on covalent bonds that are cleaved after polymerization and must be regenerated again during the recognition process. Despite the higher selectivity of such a system, finding the reversible covalent bonds restrict this approach to specified templates and functional monomers [6]. In early 1980s, non-covalent approach was introduced and is still the most common synthesis method due to its inherent advantages including simpler synthesis strategy and the wide variety of commercially available functional monomers [7]. Imprinted polymer particles with different shapes and sizes can be prepared using several synthetic strategies including bulk polymerization, precipitation polymerization, emulsion polymerization, surface imprinting and solid-phase imprinting. The fundamental principles of MIP-technology can be found in other published review manuscripts [8,9,10,11,12]. Since its introduction over thirty years ago, imprinted polymer materials have been used for a wide variety of applications [13,14,15,16]. However, MIPs are commonly used as selective sorbent materials: (I) to develop new selective sample-preparation methods (e.g., solid-phase extraction, solid-phase microextraction, etc.) for various target molecules/ions [17,18,19,20,21,22] and (II) to modify different sensor devices [23,24,25,26].

**Figure 2:**
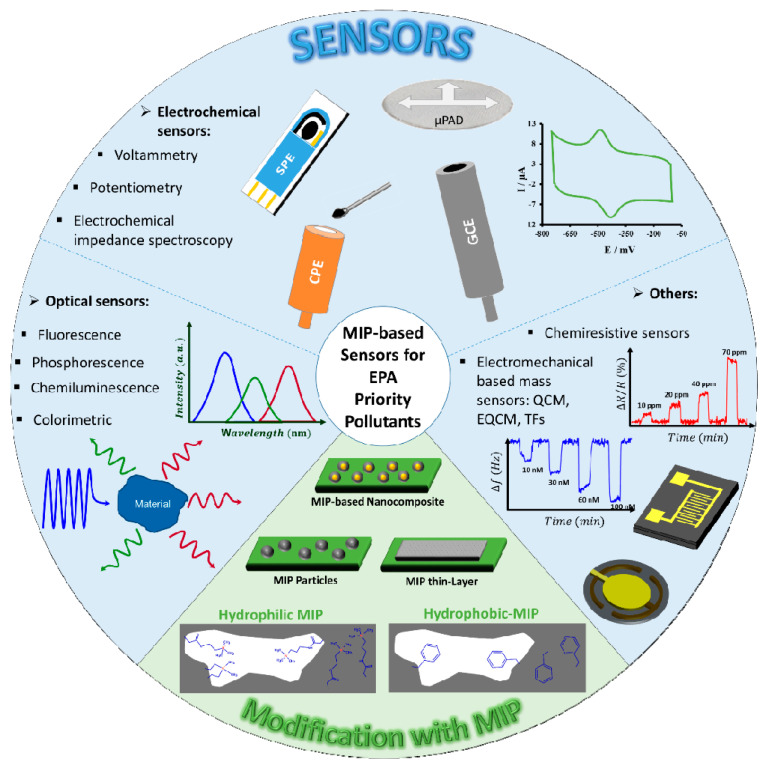
MIP-modified sensors for EPA Priority Pollutants.

As for the EPA Priority Pollutants, MIPs have been also used to develop new selective sample-preparation methods [27,28,29] or to modify different sensor devices (i.e., MIP-modified sensors: it refers to both sensor devices and their modification with MIP materials). The functionality of the used sensors is mainly based on the optical (fluorescence [30,31,32,33,34,35,36,37,38,39,40,41,42,43,44], phosphorescence [45,46,47,48], chemiluminescence [49] or color-change [50,51]) and electrochemical (electrochemical impedance spectroscopy [52], potentiometric [53,54] or voltammetric methods [55,56,57,58,59,60,61,62,63,64,65,66,67,68,69,70,71,72,73,74]) detection techniques. However, to a lesser extent, electromechanical-based mass sensors (quartz crystal microbalance [75,76,77,78,79], electrochemical quartz crystal microbalance [80] and quartz crystal tuning fork devices [81]) and chemiresistive sensors [82,83,84] were also modified with MIPs for selective detection of EPA priority pollutants. Figure 2 shows schematically the MIP-modified sensors for EPA Priority Pollutants.

An important aspect in the fabrication of sensing devices is low-cost manufacturing and thus low purchasing cost and low-cost of operation. Disposable sensors are such low-cost devices which are intended to be used by non-experienced users for the simple monitoring of target molecules, biomolecules and ions [85]. Electrochemical, colorimetric and chemiresistive sensors can be developed as low-cost devices, which makes them suitable candidates for different applications as disposable sensors. However, these sensors are mostly suffer from the low selectivity. Additionally, colorimetric and chemiresistive sensors have generally low sensitivity. In order to increase the selectivity and/or sensitivity of the sensor devices, the most widely used strategy is their modification with different recognition elements including bioreceptors like antibodies and bio(mimetic) receptors like MIPs [85]. Fluorescence, chemiluminescence and electromechanical sensors are sensitive and relatively fast but the required accessories are somewhat expensive, bulky or fragile which make it difficult to use them by a non-experienced user. In recent years, these types of sensors have shown a considerable growth as disposable sensors, supported by the impressive technological advances in different fields of engineering.

Nearly all of the developed MIP-modified sensors have been used for the selective and sensitive detection of EPA Priority Pollutants in the liquid samples. In the gaseous samples, the spatial configuration of imprinted recognition sites changes, when they are dried in air. Additionally, in gaseous samples, mass-transfer of the template molecules between the sample matrix and the imprinted recognition sites within the highly cross-linked polymer network is low and resulted, mostly, to a prolonged sampling time. There are some reports in which MIP-modified sensors were used for detection of benzene (boiling point: 80.81 °C; vapor pressure: 94.8 mm Hg at 25 °C) [78,79,81], toluene (boiling point: 111 °C; vapor pressure: 28.4 mm Hg at 25 °C) [54,77,79,81,84], xylenes (boiling points for *ortho*-: 144 °C, *meta*-: 139 °C and *para*-: 138 °C; vapor pressures at 20°C for *ortho*-: 7 mmHg, *meta*-: 9 mmHg and *para*-: 9 mmHg) [77,79,81], nitrobenzene (boiling point: 211 °C; vapor pressure 0.245 mm Hg at 25 °C) [83] and 2,4-dinitrotoluene (boiling point: decomposes at 250–300 °C; vapor pressure: 1.47 × 10^−4^ mm Hg at 22 °C) [44] in the gaseous samples. These chemicals belong to either the group of very volatile organic compounds (VVOCs) or volatile organic compounds (VOCs), which can be present with higher concentrations in air. However, MIP-modified sensors for gaseous samples suffers generally from the prolonged sampling time. In contrast to VVOCs and VOCs, semi volatile organic compounds (SVOCs) have generally lower volatility, which restricts their concentrations in air [86,87]. Therefore, SVOCs detection in gas phase is still a great challenge. In addition to the organic molecules, important metal ions are also included in the EPA Priority Pollutant List. In order to monitor the quality of water samples, different sensors were modified with ion imprinted polymers (IIPs) for sensitive and selective detection of metal ions including arsenic [88,89], mercury [90], cadmium [91,92], chromium [93,94], copper [95,96], lead [97,98], silver [99], thallium [100] and zinc [101]. However, IIPs and metal ions are not in the scope of this review and are not discussed here.

The most important feature of an imprinted polymer is its selectivity towards a target or a group of chemical compounds. Concerning selectivity assessment of the synthesized imprinted polymers, either (I) equilibrium rebinding experiments was investigated or (II) sensor signal towards the target molecule and its structurally related analogs were evaluated. Imprinting factor is another important feature, which shows the efficiency of synthesis procedure for imprinting the polymer. The imprinting factor is also reported by comparing the MIP-modified sensor signal with a sensor, which is modified with non-imprinted polymer (NIP).

Recently, review manuscripts have been published, describing imprinted polymers in combination with sensor devices that work based on a specific detection technique like electrochemical [102,103] or optical methods [104,105]. This review aims to provide an overview of the developed strategies for modification of sensor devices, that work based on different detection techniques (including optical, electrochemical, electromechanical and chemiresistive sensors), with MIPs (MIP particles or MIP thin layers were used either directly or in a nanocomposite) for a broad range of important chemicals with different physical-chemical properties. To this aim, EPA Priority Pollutant List (Table S1) was used as a chemical list that contains accepted priority pollutants and all the modified sensor devices with MIPs for these chemicals are reviewed.

## 2. Optical Sensors

An optical sensor converts electromagnetic waves into electrical signals. In MIP-modified sensors for EPA Priority Pollutants, conversion is often based on fluorescence, phosphorescence, chemiluminescence or color-change.

### 2.1. Fluorescence Based Sensors

**Figure 3:**
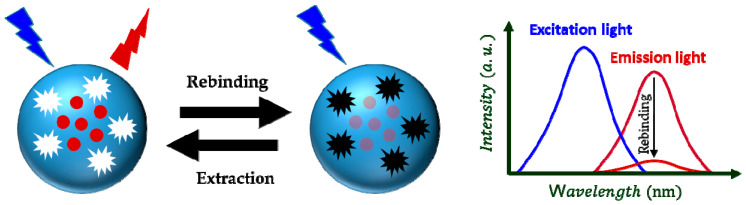
The basic working principle of the most MIP-based fluorescence sensors.

The basic working principle of the most MIP-based fluorescence sensors is quenching of a fluorophore in the presence of the target chemical compound (Figure 3).

Various fluorophores were used to develop MIP-modified fluorescence sensors including quantum dots (QDs), fluorescent organic molecules within the polymer matrix or the target molecule itself. In order to make a sensitive fluorescence-based MIP-modified sensor, the background noises must be mitigated. This was evaluated by a Monte Carlo model using anthracene-imprinted polyurethane films having various thicknesses [106]. Developed model suggests that the imprinted polymers would need to have reduced absorption coefficients or to have a quantum yields much lower than that of the analyte at the detection wavelength. In another study, fluorescence lifetimes and anisotropies were evaluated to distinguish between the analyte and the imprinted polymers when their fluorescence spectra overlap [107].

Fluorescence technique is an excellently sensitive sensing concept and can be influenced not only by quenching but also by excimer formation. This was evaluated by comparing the functionality of a fluorescence sensor with a quartz crystal microbalance (QCM) sensor using the polyurethane which was imprinted with binary templates [108]. Imprinting the polymer with two template PAHs has turned out to increase the sensors sensitivity. The sensitivity of fluorescence sensor was greater than QCM sensor; however, the signal was saturated at lower concentration, due to the formation of excimers (approximately 15 μg L^−1^ for fluorescence and 60 μg L^−1^ for QCM).

Semiconductor quantum dots (such as Mn^2+^ doped ZnS QDs, CdSe QDs, CdTe QDs) have been frequently used as fluorophore to develop MIP-modified fluorescence sensors for EPA Priority Pollutants.

Stringer et al. [43] proposed an interesting methodology to develop a fluorescence sensor for 2,4-dinitrotoluene (2,4-DNT) using amine-functionalized CdSe quantum dots (CdSe QD-NH_2_) as fluorophore. In this study, heterogeneous imprinted polymer microparticles were synthesized using bulk polymerization in chloroform. After polymerization, the CdSe QD-NH_2_ were covalently bonded to the 2,4-DNT-imprinted polymer particles (≤20 µm). In a another study by the same group [42], polymer particles were synthesized in chloroform and water, as porogen, using precipitation polymerization. Synthesized polymer particles were labeled with QDs, as described in their previous study. The fluorescence signals were used to illustrate which porogen, chloroform or water, provided more efficient MIP particles. Obtained results proved that the synthesized polymer in chloroform had greater imprinting efficiency and higher template rebinding than those prepared in water [42]. The obtained limit of detection was improved from 30.1 µM [43] to 10 µM [42] due to a new morphological structure which was obtained using precipitation polymerization. Unfortunately, the obtained sensitivities and linear ranges were not reported.

In both of the previously mentioned manuscripts, QDs were covalently bonded to the as-prepared imprinted polymer particles. Encapsulation of QDs during polymerization is another strategy which is used more often to develop fluorescence sensors. In a series of studies [40,41], QDs were embedded within the imprinted polymers to develop fluorescence sensors for dibutyl phthalate (DBP). In the first study [41], Mn-doped ZnS QDs (ZnS:Mn QDs) were synthesized and coated with a thin silica-shell using 3-mercaptopropyltriethoxysilane (MPTS). The as-prepared MPTS-capped-ZnS:Mn QDs (average diameter: ≈4 nm) were coated with imprinted layer using DBP as the template molecule, 3-aminopropyltriethoxysilane (APTES) as the functional monomer and tetraethoxysilane (TEOS) as the cross-linker. Rebinding of the template molecules decreased the emission wavelength (λem ≈ 610 nm) of embedded QDs (the excitation wavelength, λex, was set at 342 nm). The imprinting factor of 3.56 was calculated by dividing the Stern–Volmer constants (K_sv_: it is the product of the quenching rate constant and the fluorescence lifetime in absence of added quencher [109]), which were obtained for MIP- and NIP-modified sensors (Table 1). In another study by the same group [40], silicon dioxide (SiO_2_) nanoparticles (diameter: 200–300 nm) were synthesized and modified with vinyl groups using 3-(methacryloyloxy)propyltrimethoxysilane (KH-570). The vinyl functionalized SiO_2_ nanoparticles were coated with a thin imprinted polymer layer (thickness: 5 nm) by precipitation polymerization using DBP as the template molecule, acrylamide (AM) as the functional monomer and ethylene glycol dimethacrylate (EGDMA) as the crosslinker in the presence of vinyl-functionalized ZnS:Mn QDs (diameter: ≈4 nm). The prepared SiO2@QDs@MIPs were characterized and used as fluorescence sensor for DBP detection in water sample. The imprinting factor was calculated at 2.09, which was lower than the previous study. However, the LOD was improved due to higher sensitivity (Table 1).

Chang et al. synthesized the magnetized Fe_3_O_4_-CdTe@SiO_2_ microspheres and coated with imprinted polymer [39]. To this aim, Fe_3_O_4_ nanoparticles and CdTe QDs were separately synthesized and embedded within the SiO_2_ microspheres (Ø: 695 nm). Afterwards, the surface of prepared Fe_3_O_4_-CdTe@SiO_2_ microspheres were functionalized with vinyl groups using KH-570. The vinyl functionalized microspheres were coated with imprinted polymer layer (thickness: 26 nm) using 4-nitrophenol (4-NP) as template molecule, methacrylamide as functional monomer and EGDMA as crosslinker. In this study, the surfaces of synthesized Fe_3_O_4_ nanoparticles and QDs were modified with oleic acid, before their embedment within the SiO_2_ microspheres. This modification helped to prevent the aggregating of nanoparticles and to retain both the superparamagnetic properties and fluorescence intensity of prepared Fe_3_O_4_-CdTe@SiO_2_.

Fluorescence reduction happens when QDs are embedded within a matrix like silica. To mitigate this negative effect and increase the sensitivity, a thin MIP shell was directly synthesized at the surface of QDs. To this aim, thioglycollic acid (TGA)-modified CdTe QDs were used as the core and coated with 4-NP imprinted silicone layer using APTES as monomer and TEOS as crosslinker [38]. In another study, CdTe QDs were first synthesized in the presence of TGA and coated with a thin imprinted organic shell (~4 nm) [37]. In this study, attached TGA molecules at the surface of QDs provided carboxyl groups which were used for electrostatic interaction with 2-aminoethyl methacrylate hydrochloride (AMA), as a polymerizable surfactant (the AMA provided the vinyl group on the QDs surface). Afterwards, the thin imprinted shell was synthesized at the surface of vinyl-modified QDs by facile free radical polymerization process using 4-NP as template, AM as functional monomer and N,N’-methylenebisacrylamide (MBA) as cross-linker. Obtained sensitivities in both studies [37,38] were substantially increased (Table 1).

Most of the traditional semiconductors QDs contain heavy metals, which are known as environmental pollution with high degree of toxicity. Therefore, carbon dots (CDs) have been frequently used as the eco-friendly substitutes. Polyamine-functionalized CDs (Ø: ~4.5 nm) were firstly synthesized by a hydrothermal process using citric acid as the carbon source and poly(ethyleneimine) as the surface modifier. The amino-modified CDs were then embedded within the imprinted particles (Ø: ~20 nm) using 4-NP as template, APTES as functional monomer and TEOS as crosslinker [36]. In another study, silica-coated graphene quantum dots (GQDs), as an ecofriendly fluorescent substrate, were hydrothermally synthesized (average size of 25 nm) and were modified with MIP layer (MIP-coated GQDs ~ 40–50 nm) via the sol-gel polymerization process using again 4-NP as template, APTES as functional monomer and TEOS as crosslinker [35]. Using graphene helped to increase the sensors sensitivity, however, the obtained LOD was not improved (Table 1).

Like CDs, the semiconductor ZnO is an eco-friendly material with high chemical stability. ZnO QDs (Ø: 9.5–15.4 nm) were used as core and coated with imprinted silicone layer (40 nm) using 2,4,6-trichlorophenol as template, APTES as functional monomer and TEOS as crosslinker [34]. In another study, the sensor sensitivity was substantially increased using ZnO nanorods and their surface modification with a very thin MIP layer [33]. In the proposed strategy, ZnO nanorods (the length: 80–100 nm and the diameter: 8.5–12.5 nm) were synthesized and endowed with vinyl-groups using KH-570. The vinyl-modified ZnO nanorods (as the solid supports and optical materials) were then coated with a thin layer of 4-NP imprinted polymer (thickness: ~2–3 nm) using methacrylic acid (MAA) as the functional monomer and EGDMA by the precipitation polymerization method [33].

In addition to the semiconductors QDs and carbon-based QDs, other nanoparticles have been also used as fluorophore to develop fluorescence sensors for EPA Priority Pollutants.

LaVO_4_:Eu^3+^ nanoparticles, as fluorophore (red emission; λ_em_/λ_ex_ = 620 nm/254 nm), and magnetic Fe_3_O_4_ nanoparticles were encapsulated within a phenanthrene imprinted polymer nanocomposite (with an average size of 90 nm) using a facile ultrasonication emulsion strategy [32]. The strong fluorescence of the imprinted nanocomposite was quenched by not only the phenanthrene but also other evaluated PAHs. It is claimed that the magnetic separation of the nanocomposites from the mixture containing phenanthrene and other PAHs, prior to the luminescence detection, could help to enhance the selective fluorescence quenching of the imprinted nanocomposite for phenanthrene as template and target molecule.

YVO_4_:Eu^3+^ nanoparticles were used as the reference fluorophore in a ratiometric fluorescent sensor for detection of 4-NP in water samples [31]. In the proposed method, the sol-gel process was carried out in the presence of YVO_4_:Eu^3+^ nanoparticles as the reference fluorophore, CDs as the target sensitive fluorophore and 4-NP as template molecule. The ratiometric fluorescence technique helped to reduce some important interfering factors, e.g., the environmental effects and the probe concentration.

In addition to the previously mentioned fluorophore nanoparticles, the target molecule or an organic molecule within the polymer matrix can be also used as fluorophore. For example, electron-rich fluorescent conjugated-polymers can be imprinted for the electron-deficient targets like nitroaromatic compounds. Selective rebinding of the template molecules causes a noticeable selective fluorescence quenching of the polymer. Accordingly, a conjugated polymer-based selective sensor was developed for fluorescence detection of 2,4-DNT in gaseous phase [44]. To this aim, poly(fluorene- co-benzamide) (PFB) was bonded to cellulose nanofibril film with Suzuki-coupling grafting-to technique. -NH_2_ groups of benzamide within the backbone of PFBs were used as functionalities to bind template molecules, via hydrogen bonds, within the grafted layer. Finally, glutaraldehyde was used to crosslink the remaining –NH_2_ groups, producing imprinted cavities within the grafted layer. 2,4-DNT imprinted film provided a K_sv_ towards 2,4-DNT (3.7 × 10^−2^ s^−1^) which was 26, and 31 times larger than that towards trinitrotoluol (1.4 × 10^−3^ s^−1^) and picric acid (1.2 × 10^−3^ s^−1^), respectively. However, the developed sensor was not further evaluated to calculate the other figures of merit like the LOD and linear range.

Excitation and emission of the targets were also used to monitor the target molecules. In this regard, a transparent and hard imprinted polymer was synthesized via bulk polymerization using toluene as template and chloroform as porogen [30]. The polymer was milled and sieved to be placed in the sample compartment of a luminescence spectrometer and a flow injection analysis system was used for the analytical evaluation of the polymers. Synthesized polymer was implemented as a fluorescence optosensor for the screening of toluene, ethylbenzene and xylenes (TEXs) in drinking water samples. The fluorescence measurements were carried out at λ_ex_/λ_em_ = 260/284 nm, which correspond with the maxima excitation and emission wavelengths of TEXs. Unfortunately, LODs and linear ranges were not reported for this study.

### 2.2. Phosphorescence Based Sensors

In comparison to the fluorescent method, phosphorescent detection is known to have advantageous characteristics, including higher selectivity as phosphorescence is a less usual phenomenon than fluorescence, wider separation between the excitation and emission spectra, longer lifetimes of the emission and therefore, the possibility of interferences mitigation using an appropriate delay time [110]. In a proof-of-concept study, a room temperature phosphorescence (RTP) sensing system was developed for fluoranthene by synthesizing the iodinated MIP using an iodinated monomer [110]. Adding iodine heavy atoms to the polymer structure induced an efficient RTP emission from the analyte, once recognized by the MIP in a flow system. Building on this work and in another manuscript [48], developed RTP sensor was used for detection of fluoranthene in real water samples. This sensing concept was used in another study [47] to develop an RTP sensor for benzo[a]pyrene detection in water samples.

Besides PAHs as target molecules, RTP sensing system has been also developed for chlorophenol compounds. For 2,4-dichlorophenol detection [46], ZnS:Mn QDs were used as they re-emit both fluorescence and phosphorescence emissions. Silica nanoparticles and ZnS:Mn QDs were synthesized and endowed with vinyl groups using KH-570. The surface of silica nanoparticles (Ø: 200–300 nm) were coated with thin MIP layers (45, 60 and 72 nm) using AM as functional monomer, EGDMA, as cross-linking agent, 2,4-dichlorophenol as template and KH-570–ZnS:Mn QDs as the assistant monomer. Nearly the same procedure was used to develop a phosphorescence sensor for 2,4,6-trichlorophenol detection in water samples [45]. In this new study, magnetic nanoparticles (400–600 nm) were coated with 2,4,6-trichlorophenol imprinted polymer layer (45–60 nm) doped with ZnS:Mn QDs. Developed strategy provides not only a sensitive sensor but also a magnetic molecularly imprinted phosphorescence composite which can be simply separated from the complex environmental samples using an external magnet.

### 2.3. Chemiluminescence Based Sensor

In addition to the fluorescence and phosphorescence conversion principles, chemiluminescence (CL) was also combined with MIP technology for selective and sensitive 4-NP detection in tap water [49]. In this study, ZnS:Mn QDs were preliminary synthesized which was capped with MPTS. Afterwards, the MPTS capped QDs were coated with MIP layer via the sol-gel polymerization process using 4-NP as template, APTES as functional monomer and TEOS as crosslinker. 4-NP could be detected easily and rapidly by the fluorescent system. However, authors used the H_2_O_2_-NaIO_4_ chemiluminescence system, as a popular CL reaction model, to combine the good selectivity of MIP with the high sensitivity of CL. To develop this approach, an interesting CL system was designed to mix systematically the selective particles, samples and CL chemicals. MIP-capped ZnS:Mn QDs inside water were first mixed with sample, then was mixed with NaIO_4_ and entered the flow-cell to react with H_2_O_2_. The CL intensity of this system was enhanced obviously by adding both MIP- and NIP-capped ZnS:Mn QDs. However, the MIP-capped ZnS:Mn QDs have a greater enhancement than the NIP ones. Despite the relatively complex process, the sensitivity of the developed sensor for 4-NP detection was not significantly improved.

### 2.4. Colorimetric Based Sensors

A colorimetric sensor converts the specified electromagnetic waves that can be perceived by the human eye as detector (color-change). A photonic crystal based colorimetric sensor was designed for 4-NP detection in water sample using molecularly imprinted polymer colloidal array (MICA) [51]. The sensing assay was prepared by the self-assembly of 4-NP imprinted colloidal spheres (diameter: 200 ± 5 nm) on a glass substrate in a 3-D ordered opal crystalline colloidal array structure. Afterwards, the MICA was transferred onto an adhesive tape to conserve the intact opal structure. According to the Bragg-Snell law, photonic crystals reflect certain wavelengths of light depending on the angle of incident light, the average refractive index, and structural spacing of nanostructure constituents of photonic crystals. Developed sensor provided a red-shifted more than 50 nm in response to 30 mM of 4-NP. In another work [50], imprinted polymer particles (ø 210 nm) were synthesized using emulsion polymerization and used for the fabrication of A colorimetric array sensor was developed for visual detection of nitroaromatic molecules including 2,4-DNT. This type of colorimetric sensor is a good candidate for in-field application and point-of-care testing; however, suffers from the low sensitivity. Nowadays, due to the introduction of diverse and vast amount of chemical compounds into the environment, its protection needs permanent monitoring. This issue is more critical in developing countries where equipped laboratories are also less established. Therefore, development of the cost-effective and field-portable point-of-care testing methods is necessary especially for monitoring of environmental water samples [6].

Table 1 summarizes the analytical characteristics of various optical MIP-based sensors for EPA Priority Pollutants. Semiconductor quantum dots are the most used fluorophore for development of fluorescence sensors. They are either covalently attached to the previously synthesized polymer particles or embedded within the polymer particles during the synthesis procedure. Obtained results proved that the reduction of polymer particle sizes or layer thicknesses improved the accessibility of the majority of imprinted cavities, and therefore increased the polymer capacity. Among the used semiconductor quantum dots, ZnS:Mn QDs have been frequently used for development of both fluorescence and phosphorescence sensors as they re-emit fluorescence and phosphorescence emissions. Despite the known advantageous characteristics of the phosphorescence sensors, the number of reported papers are lower than the published works for fluorescence sensors, perhaps due to the more commercially available fluorophores that emit fluorescence emission.

Unlike the complex chemiluminescence system that was developed for 4-NP detection in water sample, the developed colorimetric sensor was simple and an ideal candidate for the field applications.

## 3. Electrochemical Sensors

Electrochemical sensors provide various cost-effective and simple sensing platforms, which are relatively easy to modify with selective sorbent materials. MIP-modified electrochemical sensors were developed for EPA Priority Pollutants using different electrochemical detection techniques including electrochemical impedance spectroscopy, potentiometric and voltammetric methods. Selectivity of the developed sensors was substantially increased by imprinted polymers and, in some cases, carbon-based or metal-based nanoparticles were added to improve the specific surface area and conductivity of the sensor surface. Electrochemical sensors are generally developed for detection of targets in liquid samples. However, there is one report in which a potentiometric gas sensor was developed for determination of neutral molecules of toluene in the gas phase using ion-selective electrode (ISE) [54]. In this study, an ISE membrane was impregnated with toluene-imprinted polymer particles and incubated in a gas chamber containing toluene vapor. Developed sensor was not sensitive, but the obtained LOD was low-enough for detection of toluene in gaseous samples. In another interesting study [53], potentiometric technique was used in a self-powered sensor for detection of 4-NP in water samples. In this novel approach, 4-NP imprinted polymer was used to increase the selectivity of a photocathode-based photocatalytic fuel cell (PFC) [53]. Usually, a PFC consists of a photoanode for the oxidation of fuel under photoirradiation and a noble metal cathode for catalytic reduction of an electron acceptor. In this study, the modified glassy carbon electrode (GCE) with p-type PbS quantum dots and 4-NP imprinted polymer served as the photocathode for the reduction of 4-NP under photoirradiation. On the other side, a graphene-modified GCE was employed as the anode for the oxidation of ascorbic acid. Developed sensor was stable (~30 min) and showed a fast response (~20 s) with LOD of 0.031 µM towards 4-NP.

As mentioned, most of the designed MIP based electrochemical sensors have been used for detection of EPA Priority Pollutants in liquid samples. Tap water and environmental river water samples are among the most used real samples for validation of developed sensors. Among all electrochemical detection techniques, voltammetric methods have been frequently applied in combination with different MIP-modified electrodes. In recent years, in order to enhance the sensing functionality of MIP based electrochemical assays, a large spectrum of nanostructured materials such as graphene, carbon nanotubes, and metal nanoparticles have been incorporated to imprinted polymers for the surface modification of GCE.

Graphene is known to has an ultrahigh specific surface area with a theoretical value of 2630 m^2^ g^−1^ [74]. Surface modification of graphene with thin MIP layers can help to provide a high loading capacity and increased accessibility with improved kinetics. Liu et al. [74] modified a GCE with a MIP-graphene oxide (MIP-GO) nanocomposite and used as an electrochemical sensor for 2,4-dinitrophenol detection in water sample. The amino-functionalized graphene oxide particles were simply added to the polymerization mixture containing 2,4-dinitrophenol as template and o-phenylenediamine (o-PD) as both a functional monomer and a cross-linker. The prepared GO-MIP was dispersed in water, dropped and casted on the surface of the cleaned GCE. GO-MIP/GCE provided certainly more sensitivity and selectivity in comparison to GO-NIP/GCE. However, the obtained LOD at 0.4 µM for GO-MIP/GCE is relatively high which shows that the implementation of nanomaterials like graphene does not guarantee the higher sensitivity.

In another study, an electrochemical sensor for 4-NP detection was developed by surface modification of a GCE with MIP-reduced graphene oxide (MIP-rGO) nanocomposite using drop-casting method [73]. First, rGO was functionalized by non-covalently attachment of 4-vinylcarbazole onto the surface of rGO via π-π interaction. Then, imprinted polymer layer at the surface of vinyl group functionalized rGO was synthesized (MIP/rGO, thickness: 35 nm) using 4-NP as template, MAA as monomer and EGDMA as cross-linker in a simple precipitation polymerization condition.

Using another synthesis strategy, a new MIP/rGO nanocomposite was prepared and used to modify a GCE for electrochemical detection of 4-NP [72]. The prepared rGO through the incomplete reduction of GO was coated with 4-NP imprinted polymer layer by the preliminary surface modification of rGO with 4-NP molecules via hydrogen bond and π-π interactions. Both developed sensors in [73] and [72] enabled sensitive detection of 4-NP with a LOD at 5 nM. However, the developed sensor in [73] provided two linear ranges up to 1000 µM.

For 2,4-dichlorophenol detection, two electrochemical sensors were developed using GO [71] and rGO [70]. In the first study, GCE was modified with GO and then a dispersion of 2,4-dichlorophenol imprinted polymer particles in an organic solvent was drop casted on the surface of a GO/GCE [71]. In the other study, GCE was first modified with polydopamine-rGO (PDA-rGO) [70]. Then the PDA-rGO/GCE was further modified with imprinted polymer via electropolymerizing of o-PD, as functional monomer, in the presence of 2,4-dichlorophenol as template molecule. It is assumed that the hydroxyl groups and the benzene rings of PDA-rGO could attract positive charged o-PD and also provide π-π stacking effect with o-PD and 2,4-dichlorophenol, which could make the compact imprinted film and more imprinted sites. In this study, reduction of the relative current intensity of ferro/ferricyanide was used as a probe for indirect detection of 2,4-dichlorophenol. The signal was decreased as more target molecules were adsorbed during 6 min sampling procedure. Both developed sensors, using GO [71] and functionalized rGO [70], reported nearly the same LODs (Table 2). However, the obtained sensitivity for the functionalized rGO-modified electrode was much more than the GO-modified electrode. That could be attributed to the different electrode surface area which was not considered for sensitivity calculation.

Nie et al. [69] proposed a selective electrochemical sensor for 2,4-DNT based on a composite of multiwalled carbon nanotubes (MWCNs) and MIP. The surfaces of MWCNs were preliminary modified with vinyl and amine groups, using a relatively complicated procedure. Afterwards, 2,4-DNT imprinted polymer was synthesized at the surface of MWCNs. MIP-modified MWCNs were mixed with chitosan solution and coated at the surface of GCE. Developed sensor enabled sensitive detection of 2,4-DNT with a LOD at 0.001 µM; however, the preparation procedure was complex.

Besides the carbon nanomaterials, the incorporation of metal nanoparticles into MIP improves the efficiency of the electrochemical MIP-based sensors. Liu and coworkers fabricated an electrochemical sensor for detection of 2,4-dichlorophenol in water samples based on a modified GCE with Fe_3_O_4_ nanoparticles and MIP [68]. After the preliminary modification, the surface of Fe_3_O_4_/GCE was coated with imprinted polymer via electropolymerizing of pyrrole, as functional monomer, in the presence of 2,4-dichlorophenol as template molecule. Modified sensor enabled detection of 2,4-dichlorophenol at a linear range of 0.04–2.0 μM, with a detection limit of 0.01 μM.

Ni fiber enables an enhanced electrocatalytic activity for oxidation of organic compounds, which helps to increase the sensitivity of the electrochemical method. Therefore, Ni fibers and hydrophilic 2,4-dinitrophenol imprinted polymer particles were synthesized and used for modification of a GCE using drop-casting method [67]. This helped to reduce the LOD to 0.00054 µM, in comparison to the obtained 0.4 µM in [74].

After GCE, carbon paste electrode (CPE) is another carbon-based electrochemical electrode, which is modified with imprinted polymer particles or a selective nanocomposite.

Alizadeh et al. developed the first MIP-modified CPE using 4-NP imprinted polymer which was synthesized according to the simple precipitation procedure [66]. In order to prepare the sensor, homogenized graphite, polymer powder and n-eicosane (as binder) were mixed and the prepared paste was used to fill a hole at the end of an electrode body.

CPE was also modified with a selective 4-NP imprinted polyaniline-GO nanocomposite for electrochemical detection of 4-NP in water samples [65]. In order to prepare the nanocomposite, aniline as monomer was polymerized in the presence of 4-NP as template molecule on GO sheets using ammonium persulfate as initiator by precipitation polymerization method. The synthesized MIP/GO was used in combination with graphite and paraffin to prepare a modified CPE. Comparison of the modified CPEs with polymer nanoparticles [66] and MIP-GO nanocomposite [65] show that the nanocomposite provided a wide linear range from 60 to 140 µM, while polymer nanoparticles enabled 4-NP detection in lower concentrations (Table 2).

In another study, a new electrochemical molecularly imprinted polymer (e-MIP) was synthesized and used to modify a carbon paste electrode for electrochemical detection of non-electroactive benzo[a]pyrene [64]. The microsized e-MIP beads (from 1.5 to 2.4 µm) were synthesized by copolymerization of vinylferrocene (VFc), as functional monomer and redox tracer, with EGDMA as crosslinker in the presence of benzo[a]pyrene as template molecule using distillation–precipitation polymerization method. Despite the interesting synthesis strategy, developed sensor (with LOD: 0.09 µM) needed a prolonged incubation time (4 h) and was not used for a real sample analysis.

Building on their previous work, authors evaluated the performance of their e-MIP by using 4-vinylpyridine (4 VP) as an additional comonomer [63]. Authors reported that the additional comonomer (VFc-4 VP-EDMA) increased the adsorption capacities by a factor 6–8, in comparison to the reference polymer, which contained just vinylferrocene (VFc-EDMA). However, the imprinting factor was not increased. Despite the same prolonged incubation time (4 h), the best LOD was reported at 0.93 µM for the polymer based on VFc-EDMA.

Screen-printed electrode (SPE) is a suitable device for developing disposable sensors. Nowadays, there are different SPEs in the market having various working electrodes. However, there is just one relatively old manuscript in which a screen-printed carbon electrode (SPCE) was modified with 1-hydroxypyrene (1-OHP) imprinted polymers [62]. Besides, this modified sensor was not used for a real sample analysis. It is interesting to know that the 1-OHP is actually a hydrophilic metabolite of PAHs which is produced by addition of one or more hydroxyl groups to the parent molecules after their ingestion by organisms such as fish. In this study, imprinted polymer for 1-OHP was synthesized via traditional bulk polymerization using styrene as monomer and divinylbenzene as crosslinker (the binding was only based on hydrophobic interactions). The sieved polymer particles (53 µm sieve) were mixed with carbon ink and spread over a defined area of the SPCE. Batch binding studies revealed that the optimum uptake of 1-OHP by the MIP occurred from the solutions containing 35% water in methanol. Therefore, MIP-modified SPCE was incubated in 35% aqueous methanolic 1-OHP solutions for 1 h, rinsed with buffer and subjected to cyclic voltammetry for final detection [62]. As mentioned, different new SPEs are introduced to the market having various working electrodes like carbon electrode with microholes, gold electrode, silver electrode, etc. We believe that the modification of these low-cost SPEs with imprinted polymer nanoparticles or thin layers, using new synthesis strategies, offers a great potential to develop new selective disposable sensors for EPA Priority Pollutants detection.

Microfluidic paper-based analytical device (µ-PAD) is another suitable substrate for developing disposable sensors. A photoelectrochemical sensor was developed on a µ-PAD for the detection of heptachlor in water and milk samples [61]. Wax was used as the paper hydrophobization and insulation agent to construct the µ-PAD device containing the sample and auxiliary zones. A carbon electrode was screen-printed at the back of the sample zone to create a paper working electrode (PWE). In the paper sample zone, a layer of Au nanoparticles (AuNPs) was grown on the surfaces of the cellulose fibers to increase the conductivity of the porous structure of the paper within the sample zone (Au-PWE). Finally, an imprinted polyaniline (PANI) layer was grafted in the porous Au-PWE by electropolymerization of aniline in the presence of heptachlor as template molecule. PANI, as a conjugated polymer, is an excellent photoelectric material due to the high absorption coefficient in the visible part of spectrum, high mobility of charge carrier and excellent stability. Under visible light irradiation, electrons in the highest occupied molecular orbital (HOMO) of MIP film were excited and transferred to the lowest unoccupied molecular orbital (LUMO) of the MIP film. Then, excited electrons in LUMO were delivered to the AuNPs. In this study, ascorbic acid was used to consume the positively charged holes of the imprinted PANI in an oxidation process, therefore generating a high and stable photocurrent. However, this photocurrent decreases as the heptachlor concentration was increased. The authors speculated that the selectively adsorbed heptachlor molecules by the imprinted PANI increase the steric hindrance between the ascorbic acid molecules and the photogenerated holes on the electrode interface, and consequently decrease the photocurrent.

In another study, a photoelectrochemical sensor was developed for the selective detection of lindane in water sample [60]. To this end, a MIP thin film was electropolymerized on titanium dioxide nanotubes (TiO_2_) using o–PD as monomer and lindane as template molecule (PoPD@TiO_2_). The prepared PoPD@TiO_2_ nanotubes were used as the working electrode. The authors inferred nearly the same mechanism for the photocurrent creation as they described in their previous study [61]. However, the photocurrent was increased in the presence of lindane molecules. The authors speculated that the recognition sites on the surface of MIP film could rebind the lindane molecules, which were photocatalytically oxidized by the created positive charged holes in the MIP film and, therefore led to a larger enhanced photocurrent.

The conjugated-polymer PANI is not only an excellent photoelectric material but also an interesting substrate for fabrication of electrochemical sensors. In a study [59], polyvinyl sulphonic acid (PVSA) was added to the structure of PANI to enhance the performance of the developed MIP-modified electrochemical sensor. To this aim, the polyaniline-polyvinyl sulfonic acid composite film (PANI-PVSA) has been fabricated onto an indium tin oxide (ITO) coated glass substrate by electrochemical polymerization of aniline in the presence of PVSA as modifier and 4-NP as template. The presence of PVSA in PANI results in increased conductivity, enhanced charge transfer characteristics and stability of the PANI/ITO film.

In another study [58], the surface area of an ITO electrode was preliminary modified with a nanocomposite, containing ZnO nanoparticles/MWNTs-chitosan, to increase the active sites for the electron transfer process. In order to increase the selectivity of this electrode, imprinted sol-gel solution was prepared and electrodeposited onto the nanocomposite-modified electrode.

Gold electrode is another working electrode that has been modified with selective MIP-nanocomposites. An interesting synthesis strategy was developed for preparation of macroporous imprinted polymer layer containing gold nanoparticles at the surface of a gold electrode [57]. Developed layer was prepared through combining molecular imprinting and the layer-by-layer assembly techniques for electrochemical detection of 4-NP. To this aim, thiol- functionalized silica microspheres (Ø: 420 nm) and gold nanoparticles (Ø: ~18 nm) were assembled, layer by layer, on the surface of a gold electrode. Afterwards, this electrode was further modified with imprinted polymer by electropolymerization of pyrrole in the presence of 4-nitrophenol as template molecule. Finally, silica microspheres were removed by hydrofluoric acid to produce the macroporous structure. The macroporous structure provided an outstanding high adsorption capacity with a linear range from 0.1 to 1400 µM.

In another study, the surface of a gold electrode was modified with a selective and magnetic nanocomposite, HS-MGO@AuNPs-MIP (containing thiol functionalized magnetic GO: HS-MGO, gold nanoparticles: AuNPs and molecularly imprinted polymer: MIP), for determination of DBP in wine sample [56]. In this study, the nanocomposite was magnetized to make the preparative nanocomposites easy to be separated during the synthesis process and AuNPs were used to improve the electrical conductivity of the sensor. AuNPs were decorated of the surface of HS-MGO via a self-assembly process. Finally, imprinted polymer was synthesized at the surface of HS-MGO@AuNPs substrate using DBF as template molecule, MAA as monomer and EGDMA as crosslinker via a simple precipitation polymerization method. For electrochemical measurements, HS-MGO@AuNPs-MIP particles were mixed with chitosan solution and coated at the surface of gold electrode. In comparison to the developed fluorescence sensors [40,41], the electrochemical sensor [56] enabled DBP detection at very low concentrations with a LOD at 0.0008 µM.

Using a very interesting approach, a new gold surface with a novel lamellar-ridge architecture (lamellar ridge-Au) was prepared by standard electroless deposition using Morph butterfly-scales as architecture template [55]. This approach helped to increase the accessible surface area of the electrode. The composition of the butterfly-scales is chitin which could be degraded by rinsing in phosphoric acid. After the gold deposition process, the used chitin-based biotemplate was selectively removed. In order to increase the selectivity of prepared lamellar ridge-Au towards 4-NP, recognition sites were created at the gold surface using surface molecular imprinting method. So, gold sample was incubated in a mixed solution of 1-dodecanethiol, *p*-toluenethiol and molecular template 4-NP. Finally, template molecules were removed and modified surface was used for selective determination of 4-NP using differential pulse voltammetry. Despite the innovative method for creation of the gold substrate, the sensitivity and linear range were not significantly improved.

Electrochemical impedance spectroscopy is another electrochemical detection technique, which was used to develop an electrochemical sensor. An impedance electrochemical sensor was developed for sensitive detection of dichlorodiphenyltrichloroethane (DDT) in food samples [52]. To this aim, an imprinted polymer layer (thickness: 40 nm) was synthesized at the surface of magnetic Fe_3_O_4_ nanoparticles (diameter: 450 ± 25 nm) by self-polymerization of dopamine and bisphenol A as dummy template. The MIP modified magnetic nanoparticles were then incubated with real samples to extract DDT molecules. After 2 h incubation, loaded nanoparticles were simply separated by an external magnet, washed and dropped at the surface of a GCE. Finally, the value of the charge transfer resistance of the electrode, as sensor signal, was measured by electrochemical impedance spectroscopy.

MIP-modified electrochemical sensors provide more sensitivity and loading capacity in comparison to the MIP-modified optical sensors due to larger sensor surface areas. Additionally, interesting approaches were developed to reduce the polymer sizes and increase its porosity. The other fascinating features are their cost and simple modification procedure. Non-covalent approach was used to synthesize MIPs using different functional monomers. However, MAA and EGSMA are still the most used functional monomer and cross-linker, respectively. o-PD and aniline are other interesting functional monomers that were used to create the more hydrophilic polymers which are of great interest for water analysis. Besides monomers, glycidylmethacrylate was also used as pro-hydrophilic co-monomer to induce a hydrophilic behavior to the imprinted particles after the epoxide ring opening [67].

A list of electrochemical sensors used for the detection of EAP Priority Pollutants is presented in Table 2.

## 4. Other Sensors

### 4.1. Electromechanical Based Mass Sensors

QCM is a piezoelectric device which has been frequently modified with imprinted polymers. Fu et al. [111] evaluated, systematically, the selective adsorption of gaseous-phase small organic molecules on the MIP-modified QCM sensors. To this aim, imprinted polymers were synthesized using bulk and precipitation polymerization procedures for phenol and hydroquinone, as dummy template molecules. The prepared MIP-modified QCM sensors were used to detect toluene, benzene, trichloroethylene, carbon tetrachloride, and heptanes in the gas phase. This study was more focused on polymer evaluation rather than analytical assessments.

In a pioneering study, Matsuguchi et al. proposed an interesting imprinting strategy for toluene and *p*-xylene as a solvent and applied the polymers for QCM-based VOC vapor sensing [77]. In the new proposed strategy, functional monomer and cross-linker were dissolved in toluene or p-xylene which acted simultaneously as the solvent (porogen) and the template molecule. The MIP powders were then mixed with acetone solution of poly(methyl methacrylate) and spin-coated on the QCM surface and dried. The response of the sensor towards toluene or p-xylene vapor was reversible; however, the response time was slow due to the existence of the matrix polymer around the MIP particles (60–120 min).

In order to obtain the most sensitive modified sensor towards the vapors of aromatic hydrocarbons, Banerjee et al. [79] modified the surface of a silver-coated QCM with different polymers. Different polymerization mixtures including various template molecules were evaluated. Obtained results showed that the synthesized imprinted polymer using 1,2,3-trimethoxybenzene as a dummy template and tung oil-styrene- divinylbenzene (acting all together as porogen, monomer and cross-linker) performed as the most efficient sorbent for all of the evaluated hydrocarbons. It was found that the larger amount of MIP coating hardly couples mechanically to the QCM and the quartz crystal may permanently damage due to disproportionate mass deposition on the quartz wafer. Also, the thicker coatings of MIP on the QCM surface flake off easily, resulting in a reduced sensitivity of the sensor [79]. Analyzing the gaseous samples with MIP-modified sensors needs generally a prolonged sampling time. Interestingly, the optimized modified sensor could respond relatively fast in less than 10 s; but it was reusable after complete washing with pure ethanol. Linear calibration curves were observed at the concentration range between 5 ppm to 250 ppm for benzene (2.605 Hz/ppm), toluene (2.147 Hz/ppm) and xylenes (*ortho*: 1.695, *meta*: 1.993 and *para*: 1.645 Hz/ppm) at relative humidity of 35%. Unfortunately, the optimized sensor was just evaluated for the selected aromatic hydrocarbons and other possible interferences in a real sample analysis were not evaluated.

In order to detect the actual concentrations or ppm-level partial pressures of toxic gas mixtures using adsorption isotherms, a simple sensor array (containing two different MIP-modified QCM sensors) was developed for single and binary component systems at various pressures [78]. For this goal, two MIPs were synthesized using dummy templates and the prepared polymer particles were adhered to the surface of QCMs using polyisobutylene as a binder. The obtained results from the sensor array were used for modeling purposes and the figures of merit for the modified sensors were not reported.

Besides QCM devices, quartz crystal tuning forks (TFs; microelectromechanical resonators) have been modified with imprinted polymers for gas phase detection of pollutants. An array of microfabricated TFs were modified with imprinted polymers and coupled to a short gas chromatography (GC) column for fast (total chromatogram ~ 200 s) detection of BTX chemical compounds [81]. Despite the interesting approach, the developed method is not a good candidate for commercial purposes. In the market, there are more sensitive, simple and rugged detectors, e.g., photoionization detector (PID), which can be easily coupled to the GC column. In contrast to the MIP-modified sensors, PID is not a selective detector; however, the pre-separation by a GC column can help to add the selectivity feature to a GC-PID detector [112,113].

QCM has been also used for monitoring of pollutants in liquid samples. Hexachlorobenzene, a fungicide, was sensitively and rapidly detected in water sample using a QCM sensor which was modified with a thin MIP layer (400 nm) [76]. Polymerization mixture constituents were carefully selected to control the polymer network, to enhance the polymer interaction towards the target molecule, and to improve the adhesion of the thin film to the gold surface of QCM. Polymerization mixture was spin coated onto a 10 MHz QCM chip. Interestingly, the coating was irradiated (10 mW/cm^2^) just for 10 s and subsequently placed in dichloromethane in order to remove the template molecules. Fast polymerization (10 s) was obtained by a photoinduced cleavage of the commercial benzoin derivative (Irgacure 369) used as a photoinitiator. A long-term stability was reported for the modified sensor; however, no quantitative data was mentioned.

In another study, the surface a QCM sensor was modified with a monolayer of molecularly imprinted polymer microspheres (MIPMs) for endosulfan detection in water and milk samples [75]. Endosulfan imprinted microspheres (diameter distribution: 400–500 nm) were synthesized using the simple precipitation polymerization method. Synthesized MIPMs were mixed with polyvinyl chloride solution and spin coated onto a 5 MHz QCM chip. Developed sensor could be stored for 6 months.

Combination of QCM with electrochemical equipment forms a versatile and sensitive method which is able simultaneously to monitor either the current response or the mass-change on the surface of electrode. An interesting electrochemical quartz crystal microbalance (EQCM) device was modified with different conductive polymer layers, which were imprinted for nitrotoluene molecules including 2,4-DNT [80]. In this study, a new functional monomer, containing a phenylamine group, was synthesized and used to electropolymerize MIP-layers at the surface of the gold electrode. Using modified EQCM, 2,4-DNT could not be electrochemically detected, as the polymer layer was decomposed at the required potential (~−1.00 V) for 2,4-DNT reduction. Nevertheless, piezoelectric microgravimetry enabled the 2,4-DNT detection with a LOD at 0.76 × 10^−3^ mol L^−1^ (sensitivity ~ 1.3 Hz mM^−1^) and a linear range of 1–7 mM. Despite the observed selectivity, this sensor suffers from the low sensitivity and has not been used for a real sample analysis.

### 4.2. Chemiresistive Sensors

Chemiresistive sensors are relatively low-cost and simple devices and their performance is based on the electrical resistance/reactance change when they are in contact with target analytes. Alizadeh et al. [84] developed a chemiresistor gas sensor for toluene using a toluene imprinted polymer which was synthesized according to the Matsuguchi’s method [77]. The MIP powders were mixed with graphite in the presence of melted n-eicosane as the binder agent. The mixture was used to fill a small gap between two isolated copper rods and the resistance between the rods were measured. A linear calibration curve for toluene was obtained at the concentration range between 3.8 to 46.4 ppm (~0.35 (%∆R/R_0_) ppm^−1^) with LOD at 0.8 ppm. However, the designed sensor suffered from the long prolonged response time (60–80 min). To address this issue, they developed another chemiresistor gas sensor for nitrobenzene using a nanocomposite of polymer particles, graphene and graphite [83]. A resistive ink, containing dissolved poly(methyl methacrylate) in tetrahydrofuran and the nanocomposite, was coated on the surface of an interdigital transducer (IDT). This sensor reacted faster (~20 min) than the previously developed chemiresistor for toluene (60–80 min). It was also more sensitive (~0.51 (%∆R/R_0_) ppm^−1^) with LOD at at 0.2 ppm and a linear calibration curve at the concentration range between 0.5–60 ppm ppm. These results show that the addition of graphene could help to increase the sensitivity and decrease the response time.

A low-cost IDT was fabricated by screen-printing of interdigital electrodes (IDE) on a glass substrate and the fabricated device was coated with imprinted polyurethane layers for selective anthracene detection in water sample [82]. The thickness of the polymer layer was an essential factor, which was controlled by adjusting the rotation speed and viscosity of prepolymer solution in the spin-coating method. Increasing the thicknesses from 100 nm to 1 µm helped to increase the sensor sensitivity, however, the response times were also increased from 10–20 min (the sensors were regenerated within approximately one hour). Measurements with sensors, which were modified with thick layers (more than 5 µm), were difficult due to high resistance. On the other hand, the modified sensors with very thin layers (below than 50 nm) could not provide the reproducible results. A summary of the available electromechanical and chemiresistive sensors for EPA Priority Pollutants detection is provided in Table 3.

## 5. Conclusions and Future Perspectives

In this manuscript, MIP-modified sensors for EPA Priority Pollutants were reviewed from three different aspects including sensor devices/detection techniques, polymerization and modification methods. Mainly optical, electrochemical, electromechanical-based mass sensors and chemiresistive sensors were modified with synthesized imprinted polymer particles/layers or the selective nanocomposites containing imprinted polymers. Combination of different sensors in one sensing platform, e.g., electrochemical quartz crystal microbalance, can help to obtain the complementary chemical information in a single measurement.

Among the modified sensors, some of the electrochemical, colorimetric and chemiresistive devices were low-cost and can be used as disposable sensors. In recent years, impressive technological advances in different fields of physics and engineering: (I) have provided new low-cost sensing platforms based on e.g., metamaterials [114,115] and (II) have improved the pre-existing disposable sensors like microfluidic paper-based analytical device and screen printed electrodes. We believe that the modification of such new low-cost sensing platforms with imprinted polymer nanoparticles or thin layers, using new synthesis strategies, offer a great potential to develop new selective disposable sensors for sensitive detection of EPA Priority Pollutants and also other important target chemicals and biomolecules.

For the fabrication of MIP-based sensors, imprinted polymers were synthesized using free radical polymerization and mostly based on bulk polymerization and precipitation polymerization strategies. However, molecularly imprinted nanoparticles can be also synthesized using controlled/living radical polymerization techniques. Various selective nanocomposites were prepared by integration of imprinted polymers and different nanostructured materials like graphene, carbon nanotubes and metal nanoparticles. The combination of these nanomaterials with imprinted polymers could help, but not guarantee, to increase the sensitivity and capacity of nanocomposite. There are many other factors which control the sensing functionality of prepared nanocomposite like the types MIPs (hydrophilicity, porosity which controls capacity, etc.), the developed method for integration of the nanomaterials to MIPs and the sensor modification procedure with the prepared selective nanocomposite. Drop casting and spin coating are the commonly used methods for modification of sensors, however, a “grafting from” methodology [116] would be more beneficial to precisely modify the sensors surfaces. Development of MIP-modified sensors for detection of the targets in gaseous samples is still a challenge, due to the changes in the conformation of the binding sites when the polymer is dried. Synthesizing the MIPs for electrically charged and highly water-soluble chemical compounds is another challenge in MIP technology and must be addressed by developing new synthesis methodologies.

## Figures and Tables

**Table 1:** Analytical characteristics of developed MIP-based optical sensors for EPA Priority Pollutants. The rows of the table are arranged based on the analytes and LODs.

Target(s)	Sensor/Sample	Sensitivity	LOD	Linear Range	Analysis Time	Ref.
Toluene; Ethylbenzene; Xylenes	Fluorescence/Water	N/A	N/A	N/A	81 s	[30]
4-Nitrophenol	Fluorescence/Water	K_SV,MIP_ ~197,440 M^−1^	0.036 μM	0.5–14 µM	9 min	[33]
4-Nitrophenol	Fluorescence/Water	K_SV,MIP_ ~33,500 M^−1^	0.040 µM	1–30 µM	12 min	[38]
4-Nitrophenol	Fluorescence/Water	K_SV,MIP_ ~307,692 M^−1^	0.051 μM	0.2–8.0 μM	8 min	[37]
4-Nitrophenol	Fluorescence/Water	K_SV,MIP_ ~16,050 M^−1^	0.06 μM	0.2–50 μM	2 min	[36]
4-Nitrophenol	Fluorescence/Water	K_SV,MIP_ ~34,722 M^−1^	0.065 µM	0.14–21.6 µM	N/A	[35]
4-Nitrophenol	Chemiluminescence/Water	N/A	0.076 µM	0.1–40 µM	N/A	[49]
4-Nitrophenol	Fluorescence/Water	K_SV,MIP_ ~183,333 M^−1^	0.15 µM	0–12 μM	-	[31]
4-Nitrophenol	Colorimetric/Water	~1.6 nm mM^−1^	1 mM	1–30 mM	N/A	[51]
4-Nitrophenol	Fluorescence/Water	K_SV,MIP_ ~3000 M^−1^	N/A	0–2000 µM	N/A	[39]
2,4-Dinitrotoluene	Fluorescence/Water	N/A	10 µM	N/A	~1 min	[42]
2,4-Dinitrotoluene	Fluorescence/Water	N/A	30.1 µM	N/A	~10 min	[43]
2,4-dinitrotoluene	Fluorescence/Gas	N/A	N/A	N/A	~10 min	[44]
2,4-Dichlorophenol	Phosphorescence/Water	K_SV,MIP_ 26,910 M^−1^	0.15 μM	1.0–84 μM	40 min	[46]
2,4,6-Trichlorophenol	Phosphorescence/Water	K_SV,MIP_ 132,400 M^−1^	0.035 μM	0.1–30 µM	15 min	[45]
2,4,6-Trichlorophenol	Fluorescence/Water	K_SV,MIP_ 9230 M^−1^	0.083 μM	10–160 µM	6 min	[34]
Dibutyl phthalate	Fluorescence/Distilled spirit	K_SV,MIP_ 9160 M^−1^	0.27 µM	5–50 µM	10 min	[41]
Dibutyl phthalate	Fluorescence/Tap water	K_SV,MIP_ 29,180 M^−1^	0.04 µM	5–50 µM	33 min	[40]
Fluoranthene	Phosphorescence/Water	N/A	0.00017 µM	?–0.5 µM	N/A	[48]
Phenanthrene	Fluorescence/Milk	−95.1 ^1^	0.02 µM	0.0−33.7 µM	~90 min	[32]
Benzo[a]pyrene	Phosphorescence/Water	N/A	0.00004 µM	?–0.4 µM	N/A	[47]

N/A: not available; K_SV_: Stern-Volmer constant; CL: chemiluminescence; ^1^ (PL intensity a.u.) (mg/L)^−1^.

**Table 2:** Analytical characteristics of developed MIP-based electrochemical sensors for EPA Priority Pollutants. The rows of the table are arranged based on the analytes and LODs.

Target(s)	Electrode/Sample	Sensitivity	LOD	Linear Range	Analysis Time	Ref.
Toluene	ISE/Gas	~1.9 (μV/s) ppm^−1^	3.5 ppm	10–125 ppm	~30 min	[54]
4-Nitrophenol	ITO/Water	53.219 µA/(cm^2^ mM)	0.001 µM	0.01–200 µM	N/A	[58]
4-Nitrophenol	CPE/Water	N/A	0.003 µM	0.008–5 µM	~10 min	[66]
4-Nitrophenol	GCE/Water	0.15 µA µM^−1^	0.005 µM	0.01–100 µM	~5 min	[72]
4-Nitrophenol	GCE/Water	0.193 µA µM^−1^ 0.090 µA µM^−1^	0.005 µM	0.01–100 µM 200–1000 µM	~2 min	[73]
4-Nitrophenol	Gold/Water	1.74 mA/(cm^2^ mM) 0.58 mA/(cm^2^ mM)	0.02 µM	0.025–1 µM 1–100 µM	~10 min	[55]
4-Nitrophenol	PFC/Water	0.0295 V ^1^	0.031 μM	0.05–20 µM	~20 s	[53]
4-Nitrophenol	Gold/Water	~2.3 µA mM^−1^	0.1 µM	0.1–1400 µM	N/A	[57]
4-Nitrophenol	ITO/Water	1.5 µA µM^−1^ 50 µA µM^−1^ cm^−2^	1 µM	0–48 µM	N/A	[59]
4-Nitrophenol	CPE/Water	0.125 µA µM^−1^	20 µM	60–140 µM	N/A	[65]
2,4-Dichlorophenol	GCE/Water	1.295 µA µM^−1^ 0.05206 µA µM^−1^	0.0005 µM	0.004–0.4 μM 0.4–10.0 μM	~2 min	[71]
2,4-Dichlorophenol	GCE/Water	812.1 µA µM^−1^ 44.7 µA µM^−1^	0.0008 µM	2.0–10.0 nM 10.0–100.0 nM	~6 min	[70]
2,4-Dinitrotoluene	GCE/Water	6 µA µM^−1^	0.001 µM	0.0022–1 µM	~11 min	[69]
2,4-Dichlorophenol	GCE/Water	20.5 µA µM^−1^	0.01 μM	0.04–2.0 μM	~2 min	[68]
2,4-Dinitrophenol	GCE/Water	0.0114 mA/(µg/L) 0.161 mA/(cm^2^ µg/L)	0.00054 µM	3.8–163 nM	~3 min	[67]
2,4-Dinitrophenol	GCE/Water	0.0594 µA µM^−1^	0.4 µM	1.0–150.0 µM	~5 min	[74]
1-OHP	SPE/N/A	3.11 µA mM^−1^	N/A	0.1–1 mM	~1 h	[62]
Heptachlor	µ-PAD/Water; Milk	−3 µA nM^−1^	0.008 nM	0.03–10 nM	~250 s	[61]
Lindane	TiO_2_/Water	1.758 µA µM^−1^	0.03 µM	0.1–10 µM	~10 min	[60]
Dibutyl phthalate	Gold/Wine	1.06 µA µM^−1^	0.0008 µM	0.0025–5 µM	14 min	[56]
DDT	GCE/Food	19.33 Ω (log pM)^−1^	0.006 nM	0.01–10^6^ nM	~2 h	[52]

^1^ Slope in Nernst equation; N/A: not available; ISE: ion-selective electrode; ITO: indium tin oxide; PFC: photocathode-based photocatalytic fuel cell; GCE: Glassy carbon electrode; TiO_2_: titanium dioxide nanotubes; µ-PAD: microfluidic paper-based analytical device; SPE: screen-printed electrode; CPE: carbon paste electrode; 1-OHP: 1-hydroxypyrene (metabolite of PAHs); DDT: Dichlorodiphenyltrichloroethane.

**Table 3:** Analytical characteristics of developed MIP-based mass and chemiresistive sensors for EPA Priority Pollutants.

Sensor	Target(s)	Sample	LOD	Linear Range	Analysis Time	Ref.
QCM	Benzene; Toluene; Xylenes	Gas	Benzene (0.98 ppm) Toluene (1.25 ppm) *o*-Xylene (1.42 ppm) *m*-Xylene (1.41 ppm) *p*-Xylene (1.36 ppm)	5–250 ppm	<10 s	[79]
QCM	Benzene; IMK	Gas	N/A	N/A	~<3 min	[78]
QCM	Toluene; *p*-Xylene	Gas	N/A	N/A	60–120 min	[77]
QCM	Hexachlorobenzene	Water	10^−6^ µM	N/A	~10 s	[76]
QCM	Endosulfan	Water Milk	0.014 µM	0.025–0.1 µM 0.1–3.15 µM	400 s	[75]
EQCM	2,4-Dinitrotoluene	Water	PM: 0.76 mM	1–7 mM	10 min	[80]
TFs	Benzene; Toluene; Xylenes	Gas	Benzene (0.7 ppm) Toluene (0.23 ppm) *p,m*-Xylene (0.7 ppm)	N/A	15 to 25 s	[81]
Chemiresistor	Toluene	Gas	0.8 ppm	3.8–46.4 ppm	60–80 min	[84]
Chemiresistor	Nitrobenzene	Gas	0.2 ppm	0.5–60 ppm	~20 min	[83]
Chemiresistor	Anthracene	Water	1.3 nM	N/A	~20 min	[82]

QCM: Quartz crystal microbalance; TFs: Quartz crystal tuning forks; EQCM: Electrochemical quartz crystal microbalance; PM: Piezoelectric Microgravimetry.

## Supplementary Materials

The following are available online at https://www.mdpi.com/article/10.3390/s21072406/s1, Table S1: Published manuscripts for the chemicals in EPA Priority Pollutant List.

## Abbreviations

EPA	Environmental Protection Agency
PAHs	Polycyclic aromatic hydrocarbons
PCBs	Polychlorinated biphenyls
BTEX	Benzene, Toluene, Ethylbenzene and Xylene
DBP	Dibutyl phthalate
MPTS	3-Mercaptopropyltriethoxysilane
APTES	3-Aminopropyltriethoxysilane
TEOS	Tetraethoxysilane
KH-570	3-(Methacryloyloxy)propyltrimethoxysilane
4-NP	4-Nitrophenol
QCM	Quartz crystal microbalance
TFs	Quartz crystal tuning forks
EQCM	Electrochemical quartz crystal microbalance
µ-PAD	Microfluidic paper-based analytical device
RTP	Room temperature phosphorescence
CL	Chemiluminescence
ISE	Ion-selective electrode
GCE	Glassy carbon electrode
CPE	Carbon paste electrode
SPE	Screen-printed electrode

## References

[1] US EPA Toxic and Priority Pollutants under the Clean Water Act/US EPA. https://www.epa.gov/eg/toxic-and-priority-pollutants-under-clean-water-act.

[2] US EPA Initial List of Hazardous Air Pollutants with Modifications/US EPA. https://www.epa.gov/haps/initial-list-hazardous-air-pollutants-modifications.

[3] Wulff G. (2013). Fourty years of molecular imprinting in synthetic polymers: Origin, features and perspectives. Microchim. Acta.

[4] Wulff G. (1995). Molecular Imprinting in Cross-Linked Materials with the Aid of Molecular Templates—A Way towards Artificial Antibodies. Angew. Chem. Int. Ed. Engl..

[5] Beyazit S., Tse Sum Bui B., Haupt K., Gonzato C. (2016). Molecularly imprinted polymer nanomaterials and nanocomposites by controlled/living radical polymerization. Prog. Polym. Sci..

[6] Zarejousheghani M. (2019). Towards In-Field Sample-Preparation and Detection: Development of New Sample Preparation Formats Using Molecularly Imprinted Polymers for the Combination with Field-Deployable Detectors.

[7] Mingdi Y. *Molecularly Imprinted Materials*: *Science and Technology;* CRC PRESS: Boca Raton, FL, USA, 2020. ISBN 036757.

[8] Alexander C., Andersson H.S., Andersson L.I., Ansell R.J., Kirsch N., Nicholls I.A., O’Mahony J., Whitcombe M.J. (2006). Molecular imprinting science and technology: A survey of the literature for the years up to and including 2003. J. Mol. Recognit..

[9] Zhang H. (2020). Molecularly Imprinted Nanoparticles for Biomedical Applications. Adv. Mater..

[10] Zarejousheghani M., Lorenz W., Vanninen P., Alizadeh T., Cämmerer M., Borsdorf H. (2019). Molecularly Imprinted Polymer Materials as Selective Recognition Sorbents for Explosives: A Review. Polymers.

[11] Haupt K., Medina Rangel P.X., Bui B.T.S. (2020). Molecularly Imprinted Polymers: Antibody Mimics for Bioimaging and Therapy. Chem. Rev..

[12] O’Brien J., Shea K.J. (2016). Tuning the Protein Corona of Hydrogel Nanoparticles: The Synthesis of Abiotic Protein and Peptide Affinity Reagents. Acc. Chem. Res..

[13] Cheong W.J., Yang S.H., Ali F. (2013). Molecular imprinted polymers for separation science: A review of reviews. J. Sep. Sci..

[14] Fuchs Y., Soppera O., Haupt K. (2012). Photopolymerization and photostructuring of molecularly imprinted polymers for sensor applications-a review. Anal. Chim. Acta.

[15] Gui R., Jin H., Guo H., Wang Z. (2018). Recent advances and future prospects in molecularly imprinted polymers-based electrochemical biosensors. Biosens. Bioelectron..

[16] Ye L., Haupt K. (2004). Molecularly imprinted polymers as antibody and receptor mimics for assays, sensors and drug discovery. Anal. Bioanal. Chem..

[17] Zarejousheghani M., Fiedler P., Möder M., Borsdorf H. (2014). Selective mixed-bed solid phase extraction of atrazine herbicide from environmental water samples using molecularly imprinted polymer. Talanta.

[18] Zarejousheghani M., Möder M., Borsdorf H. (2013). A new strategy for synthesis of an in-tube molecularly imprinted polymer-solid phase microextraction device: Selective off-line extraction of 4-nitrophenol as an example of priority pollutants from environmental water samples. Anal. Chim. Acta.

[19] Zarejousheghani M., Schrader S., Möder M., Lorenz P., Borsdorf H. (2015). Ion-exchange molecularly imprinted polymer for the extraction of negatively charged acesulfame from wastewater samples. J. Chromatogr. A.

[20] Alizadeh T., Ganjali M.R., Nourozi P., Zare M. (2009). Multivariate optimization of molecularly imprinted polymer solid-phase extraction applied to parathion determination in different water samples. Anal. Chim. Acta.

[21] Zarejousheghani M., Schrader S., Möder M., Mayer T., Borsdorf H. (2018). Negative electrospray ionization ion mobility spectrometry combined with paper-based molecular imprinted polymer disks: A novel approach for rapid target screening of trace organic compounds in water samples. Talanta.

[22] Zarejousheghani M., Schrader S., Möder M., Schmidt M., Borsdorf H. (2018). A new strategy for accelerated extraction of target compounds using molecularly imprinted polymer particles embedded in a paper-based disk. J. Mol. Recognit..

[23] Alizadeh T., Ganjali M.R., Zare M., Norouzi P. (2010). Development of a voltammetric sensor based on a molecularly imprinted polymer (MIP) for caffeine measurement. Electrochim. Acta.

[24] Zarejousheghani M., Jaafar A., Wollmerstaedt H., Rahimi P., Borsdorf H., Zimmermann S., Joseph Y. (2021). Rational Design of Molecularly Imprinted Polymers Using Quaternary Ammonium Cations for Glyphosate Detection. Sensors.

[25] Alizadeh T., Ganjali M.R., Zare M., Norouzi P. (2012). Selective determination of chloramphenicol at trace level in milk samples by the electrode modified with molecularly imprinted polymer. Food Chem..

[26] Alizadeh T., Zare M., Ganjali M.R., Norouzi P., Tavana B. (2010). A new molecularly imprinted polymer (MIP)-based electrochemical sensor for monitoring 2,4,6-trinitrotoluene (TNT) in natural waters and soil samples. Biosens. Bioelectron..

[27] Deng D., He Y., Li M., Huang L., Zhang J. (2021). Preparation of multi-walled carbon nanotubes based magnetic multi-template molecularly imprinted polymer for the adsorption of phthalate esters in water samples. Environ. Sci. Pollut. Res. Int..

[28] Luo J., Gao Y., Tan K., Wei W., Liu X. (2016). Preparation of a Magnetic Molecularly Imprinted Graphene Composite Highly Adsorbent for 4-Nitrophenol in Aqueous Medium. ACS Sustain. Chem. Eng..

[29] Zuo H.G., Yang H., Zhu J.X., Ding Y. (2017). Preparation of a novel RAM-MIP for selective solid-phase extraction and gas chromatography determination of heptachlor, endosulfan and their metabolite residues in pork. Anal. Methods.

[30] Sainz-Gonzalo F.J., Medina-Castillo A.L., Fernández-Sánchez J.F., Fernández-Gutiérrez A. (2011). Synthesis and characterization of a molecularly imprinted polymer optosensor for TEXs-screening in drinking water. Biosens. Bioelectron..

[31] Li W., Zhang H., Chen S., Liu Y., Zhuang J., Lei B. (2016). Synthesis of molecularly imprinted carbon dot grafted YVO4:Eu3+ for the ratiometric fluorescent determination of paranitrophenol. Biosens. Bioelectron..

[32] Li H., Wang L. (2013). Highly Selective Detection of Polycyclic Aromatic Hydrocarbons Using Multifunctional Magnetic–Luminescent Molecularly Imprinted Polymers. ACS Appl. Mater. Interfaces.

[33] Wei X., Zhou Z., Hao T., Li H., Zhu Y., Gao L., Yan Y. (2015). A novel molecularly imprinted polymer thin film at surface of ZnO nanorods for selective fluorescence detection of para-nitrophenol. RSC Adv..

[34] Lin X., Wu Y., Hao Y., Sun Q., Yan Y., Li C. (2018). Sensitive and Selective Determination of 2,4,6-Trichlorophenol Using a Molecularly Imprinted Polymer Based on Zinc Oxide Quantum Dots. Anal. Letters.

[35] Zhou Y., Qu Z., Zeng Y., Zhou T., Shi G. (2014). A novel composite of graphene quantum dots and molecularly imprinted polymer for fluorescent detection of paranitrophenol. Biosens. Bioelectron..

[36] Hao T., Wei X., Nie Y., Xu Y., Yan Y., Zhou Z. (2016). An eco-friendly molecularly imprinted fluorescence composite material based on carbon dots for fluorescent detection of 4-nitrophenol. Microchim Acta.

[37] Yu J., Wang X., Kang Q., Li J., Shen D., Chen L. (2017). One-pot synthesis of a quantum dot-based molecular imprinting nanosensor for highly selective and sensitive fluorescence detection of 4-nitrophenol in environmental waters. Environ. Sci. Nano.

[38] Jiang L., Liu H., Li M., Xing Y., Ren X. (2016). Surface molecular imprinting on CdTe quantum dots for fluorescence sensing of 4-nitrophenol. Anal. Methods.

[39] Chang L., Chen S., Chu J., Li X. (2013). Co-assembly of CdTe and Fe3O4 with molecularly imprinted polymer for recognition and separation of endocrine disrupting chemicals. Appl. Surf. Sci..

[40] Zhou Z., Li T., Xu W., Huang W., Wang N., Yang W. (2017). Synthesis and characterization of fluorescence molecularly imprinted polymers as sensor for highly sensitive detection of dibutyl phthalate from tap water samples. Sens. Actuators B Chem..

[41] Li T., Gao Z., Wang N., Zhou Z., Xu W., Zheng J., Yang W. (2016). Synthesis and evaluation of a molecularly imprinted polymer with high-efficiency recognition for dibutyl phthalate based on Mn-doped ZnS quantum dots. RSC Adv..

[42] Stringer R.C., Gangopadhyay S., Grant S.A. (2011). Comparison of molecular imprinted particles prepared using precipitation polymerization in water and chloroform for fluorescent detection of nitroaromatics. Anal. Chim. Acta.

[43] Stringer R.C., Gangopadhyay S., Grant S.A. (2010). Detection of nitroaromatic explosives using a fluorescent-labeled imprinted polymer. Anal. Chem..

[44] Niu Q., Gao K., Lin Z., Wu W. (2013). Surface molecular-imprinting engineering of novel cellulose nanofibril/conjugated polymer film sensors towards highly selective recognition and responsiveness of nitroaromatic vapors. Chem. Commun..

[45] Wei X., Yu M., Li C., Gong X., Qin F., Wang Z. (2018). Magnetic nanoparticles coated with a molecularly imprinted polymer doped with manganese-doped ZnS quantum dots for the determination of 2,4,6-trichlorophenol. Microchim. Acta.

[46] Wei X., Zhou Z., Hao T., Li H., Xu Y., Lu K., Wu Y., Dai J., Pan J., Yan Y. (2015). Highly-controllable imprinted polymer nanoshell at the surface of silica nanoparticles based room-temperature phosphorescence probe for detection of 2,4-dichlorophenol. Anal. Chim. Acta.

[47] Traviesa-Alvarez J.M., Sánchez-Barragán I., Costa-Fernández J.M., Pereiro R., Sanz-Medel A. (2007). Room temperature phosphorescence optosensing of benzoapyrene in water using halogenated molecularly imprinted polymers. Analyst.

[48] Sánchez-Barragán I., Costa-Fernández J.M., Pereiro R., Sanz-Medel A., Salinas A., Segura A., Fernández-Gutiérrez A., Ballesteros A., González J.M. (2005). Molecularly Imprinted Polymers Based on Iodinated Monomers for Selective Room-Temperature Phosphorescence Optosensing of Fluoranthene in Water. Anal. Chem..

[49] Liu J., Chen H., Lin Z., Lin J.-M. (2010). Preparation of Surface Imprinting Polymer Capped Mn-Doped ZnS Quantum Dots and Their Application for Chemiluminescence Detection of 4-Nitrophenol in Tap Water. Anal. Chem..

[50] LU W., Dong X., Qiu L., Yan Z., Meng Z., XUE M., He X., Liu X. (2017). Colorimetric sensor arrays based on pattern recognition for the detection of nitroaromatic molecules. J. Hazard. Mater..

[51] Xue F., Meng Z., Wang Y., Huang S., Wang Q., Lu W., Xue M. (2014). A molecularly imprinted colloidal array as a colorimetric sensor for label-free detection of p-nitrophenol. Anal. Methods.

[52] Miao J., Liu A., Wu L., Yu M., Wei W., Liu S. (2020). Magnetic ferroferric oxide and polydopamine molecularly imprinted polymer nanocomposites based electrochemical impedance sensor for the selective separation and sensitive determination of dichlorodiphenyltrichloroethane (DDT). Anal. Chim. Acta.

[53] Yan K., Yang Y., Zhu Y., Zhang J. (2017). Highly Selective Self-Powered Sensing Platform for p-Nitrophenol Detection Constructed with a Photocathode-Based Photocatalytic Fuel Cell. Anal. Chem..

[54] Liang R., Chen L., Qin W. (2015). Potentiometric detection of chemical vapors using molecularly imprinted polymers as receptors. Sci. Rep..

[55] Guo X., Zhou H., Fan T., Zhang D. (2015). Electrochemical detection of p-nitrophenol on surface imprinted gold with lamellar-ridge architecture. Sens. Actuators B Chem..

[56] Li X., Wang X., Li L., Duan H., Luo C. (2015). Electrochemical sensor based on magnetic graphene oxide@gold nanoparticles-molecular imprinted polymers for determination of dibutyl phthalate. Talanta.

[57] Xu G., Yang L., Zhong M., Li C., Lu X., Kan X. (2013). Selective recognition and electrochemical detection of p -nitrophenol based on a macroporous imprinted polymer containing gold nanoparticles. Microchim. Acta.

[58] Hu Y., Zhang Z., Zhang H., Luo L., Yao S. (2012). Sensitive and selective imprinted electrochemical sensor for p-nitrophenol based on ZnO nanoparticles/carbon nanotubes doped chitosan film. Thin Solid Films.

[59] Roy A.C., Nisha V.S., Dhand C., Ali M.A., Malhotra B.D. (2013). Molecularly imprinted polyaniline-polyvinyl sulphonic acid composite based sensor for para-nitrophenol detection. Anal. Chim. Acta.

[60] Wang P., Ge L., Li M., Li W., Li L., Wang Y., Yu J. (2013). Photoelectrochemical Sensor Based on Molecularly Imprinted Polymer-Coated TiO_2_ Nanotubes for Lindane Specific Recognition and Detection. J. Inorg. Organomet. Polym..

[61] Wang P., Sun G., Ge L., Ge S., Yu J., Yan M. (2013). Photoelectrochemical lab-on-paper device based on molecularly imprinted polymer and porous Au-paper electrode. Analyst.

[62] Kirsch N., Hart J.P., Bird D.J., Luxton R.W., McCalley D.V. (2001). Towards the development of molecularly imprinted polymer based screen-printed sensors for metabolites of PAHs. Analyst.

[63] Udomsap D., Brisset H., Culioli G., Dollet P., Laatikainen K., Siren H., Branger C. (2018). Electrochemical molecularly imprinted polymers as material for pollutant detection. Mater. Today Commun..

[64] Udomsap D., Branger C., Culioli G., Dollet P., Brisset H. (2014). A versatile electrochemical sensing receptor based on a molecularly imprinted polymer. Chem. Commun..

[65] Saadati F., Ghahramani F., Shayani-jam H., Piri F., Yaftian M.R. (2018). Synthesis and characterization of nanostructure molecularly imprinted polyaniline/graphene oxide composite as highly selective electrochemical sensor for detection of p-nitrophenol. J. Taiwan Inst. Chem. Eng..

[66] Alizadeh T., Ganjali M.R., Norouzi P., Zare M., Zeraatkar A. (2009). A novel high selective and sensitive para-nitrophenol voltammetric sensor, based on a molecularly imprinted polymer-carbon paste electrode. Talanta.

[67] Jing T., Xia H., Niu J., Zhou Y., Dai Q., Hao Q., Zhou Y., Mei S. (2011). Determination of trace 2,4-dinitrophenol in surface water samples based on hydrophilic molecularly imprinted polymers/nickel fiber electrode. Biosens. Bioelectron..

[68] Liu B., Cang H., Jin J. (2016). Molecularly Imprinted Polymers Based Electrochemical Sensor for 2,4-Dichlorophenol Determination. Polymers.

[69] Nie D., Han Z., Yu Y., Shi G. (2016). Composites of multiwalled carbon nanotubes/polyethyleneimine (MWCNTs/PEI) and molecularly imprinted polymers for dinitrotoluene recognition. Sens. Actuators B Chem..

[70] Liu Y., Liang Y., Yang R., Li J., Qu L. (2019). A highly sensitive and selective electrochemical sensor based on polydopamine functionalized graphene and molecularly imprinted polymer for the 2,4-dichlorophenol recognition and detection. Talanta.

[71] Liang Y., Yu L., Yang R., Li X., Qu L., Li J. (2017). High sensitive and selective graphene oxide/molecularly imprinted polymer electrochemical sensor for 2,4-dichlorophenol in water. Sens. Actuators B Chem..

[72] Zeng Y., Zhou Y., Zhou T., Shi G. (2014). A novel composite of reduced graphene oxide and molecularly imprinted polymer for electrochemical sensing 4-nitrophenol. Electrochim. Acta.

[73] Luo J., Cong J., Liu J., Gao Y., Liu X. (2015). A facile approach for synthesizing molecularly imprinted graphene for ultrasensitive and selective electrochemical detecting 4-nitrophenol. Anal. Chim. Acta.

[74] Liu Y., Zhu L., Zhang Y., Tang H. (2012). Electrochemical sensoring of 2,4-dinitrophenol by using composites of graphene oxide with surface molecular imprinted polymer. Sens. Actuators B Chem..

[75] Liu N., Han J., Liu Z., Qu L., Gao Z. (2013). Rapid detection of endosulfan by a molecularly imprinted polymer microsphere modified quartz crystal microbalance. Anal. Methods.

[76] Das K., Penelle J., Rotello V.M. (2003). Selective Picomolar Detection of Hexachlorobenzene in Water Using a Quartz Crystal Microbalance Coated with a Molecularly Imprinted Polymer Thin Film. Langmuir.

[77] Matsuguchi M., Uno T. (2006). Molecular imprinting strategy for solvent molecules and its application for QCM-based VOC vapor sensing. Sens. Actuators B Chem..

[78] Hwang M.J., Shim W.G., Yoon S.D., Moon H. (2019). Adsorption of toxic gases on molecularly imprinted polymer coated QCM: Measurements and modeling for partial pressure in gas mixture. Adsorption.

[79] Bhattacharyya Banerjee M., Pradhan S., Banerjee Roy R., Tudu B., Das D.K., Bandyopadhyay R., Pramanik P. (2019). Detection of Benzene and Volatile Aromatic Compounds by Molecularly Imprinted Polymer-Coated Quartz Crystal Microbalance Sensor. IEEE Sens. J..

[80] Huynh T.-P., Sosnowska M., Sobczak J.W., Kc C.B., Nesterov V.N., D’Souza F., Kutner W. (2013). Simultaneous chronoamperometry and piezoelectric microgravimetry determination of nitroaromatic explosives using molecularly imprinted thiophene polymers. Anal. Chem..

[81] Iglesias R.A., Tsow F., Wang R., Forzani E.S., Tao N. (2009). Hybrid Separation and Detection Device for Analysis of Benzene, Toluene, Ethylbenzene, and Xylenes in Complex Samples. Anal. Chem..

[82] Latif U., Ping L., Dickert F.L. (2018). Conductometric Sensor for PAH Detection with Molecularly Imprinted Polymer as Recognition Layer. Sensors.

[83] Alizadeh T., Hamedsoltani L. (2014). Graphene/graphite/molecularly imprinted polymer nanocomposite as the highly selective gas sensor for nitrobenzene vapor recognition. J. Environ. Chem. Eng..

[84] Alizadeh T., Rezaloo F. (2013). Toluene chemiresistor sensor based on nano-porous toluene-imprinted polymer. Int. J. Environ. Anal. Chem..

[85] Dincer C., Bruch R., Costa-Rama E., Fernández-Abedul M.T., Merkoçi A., Manz A., Urban G.A., Güder F. (2019). Disposable Sensors in Diagnostics, Food, and Environmental Monitoring. Adv. Mater..

[86] Zarejousheghani M., Walte A., Borsdorf H. (2018). Sprayed liquid–gas extraction of semi-volatile organophosphate malathion from air and contaminated surfaces. Anal. Methods.

[87] Zarejousheghani M., Cämmerer M., Mayer T., Walte A., Borsdorf H. (2018). Sprayed liquid-gas extraction in combination with ion mobility spectrometry: A novel approach for the fast determination of semi-volatile compounds in air and from contaminated surfaces. Int. J. Ion Mobil. Spec..

[88] Alizadeh T., Rashedi M., Hanifehpour Y., Joo S.W. (2015). Improvement of durability and analytical characteristics of arsenic-imprinted polymer-based PVC membrane electrode via surface modification of nano-sized imprinted polymer particles: Part 2. Electrochim. Acta.

[89] Alizadeh T., Rashedi M. (2014). Synthesis of nano-sized arsenic-imprinted polymer and its use as As3+ selective ionophore in a potentiometric membrane electrode: Part 1. Anal. Chim. Acta.

[90] Alizadeh T., Ganjali M.R., Zare M. (2011). Application of an Hg^2+^ selective imprinted polymer as a new modifying agent for the preparation of a novel highly selective and sensitive electrochemical sensor for the determination of ultratrace mercury ions. Anal. Chim. Acta.

[91] Alizadeh T., Ganjali M.R., Nourozi P., Zare M., Hoseini M. (2011). A carbon paste electrode impregnated with Cd^2+^ imprinted polymer as a new and high selective electrochemical sensor for determination of ultra-trace Cd^2+^ in water samples. Journal of Electroanal. Chem..

[92] Huang K., Chen Y., Zhou F., Zhao X., Liu J., Mei S., Zhou Y., Jing T. (2017). Integrated ion imprinted polymers-paper composites for selective and sensitive detection of Cd(II) ions. J. Hazard. Mater..

[93] Zhang M.Y., Huang R.F., Ma X.G., Guo L.H., Wang Y., Fan Y.M. (2019). Selective fluorescence sensor based on ion-imprinted polymer-modified quantum dots for trace detection of Cr(VI) in aqueous solution. Anal. Bioanal. Chem..

[94] Lu H., Xu S. (2020). Dual channel ion imprinted fluorescent polymers for dual mode simultaneous chromium speciation analysis. Analyst.

[95] Zhihua W., Xiaole L., Jianming Y., Yaxin Q., Xiaoquan L. (2011). Copper(II) determination by using carbon paste electrode modified with molecularly imprinted polymer. Electrochim. Acta.

[96] Rajabi H.R., Zarezadeh A., Karimipour G. (2017). Porphyrin based nano-sized imprinted polymer as an efficient modifier for the design of a potentiometric copper carbon paste electrode. RSC Adv..

[97] Dahaghin Z., Kilmartin P.A., Mousavi H.Z. (2020). Novel ion imprinted polymer electrochemical sensor for the selective detection of lead(II). Food Chem..

[98] Luo X., Huang W., Shi Q., Xu W., Luan Y., Yang Y., Wang H., Yang W. (2017). Electrochemical sensor based on lead ion-imprinted polymer particles for ultra-trace determination of lead ions in different real samples. RSC Adv..

[99] Zhiani R., Ghanei-Motlag M., Razavipanah I. (2016). Selective voltammetric sensor for nanomolar detection of silver ions using carbon paste electrode modified with novel nanosized Ag(I)-imprinted polymer. J. Mol. Liq..

[100] Nasiri-Majd M., Taher M.A., Fazelirad H. (2015). Synthesis and application of nano-sized ionic imprinted polymer for the selective voltammetric determination of thallium. Talanta.

[101] Behnia N., Asgari M., Feizbakhsh A. (2015). Sub-nanomolar detection of zinc on the ion-imprinted polymer modified glassy carbon electrode. J. Environ. Chem. Eng..

[102] Wang J., Liang R., Qin W. (2020). Molecularly imprinted polymer-based potentiometric sensors. TrAC Trends Anal. Chem..

[103] Rebelo P., Costa-Rama E., Seguro I., Pacheco J.G., Nouws H.P.A., Cordeiro M.N.D.S., Delerue-Matos C. (2021). Molecularly imprinted polymer-based electrochemical sensors for environmental analysis. Biosens. Bioelectron..

[104] Ansari S., Masoum S. (2021). Recent advances and future trends on molecularly imprinted polymer-based fluorescence sensors with luminescent carbon dots. Talanta.

[105] Ma J., Yan M., Feng G., Ying Y., Chen G., Shao Y., She Y., Wang M., Sun J., Zheng L. (2021). An overview on molecular imprinted polymers combined with surface-enhanced Raman spectroscopy chemical sensors toward analytical applications. Talanta.

[106] Chen Y.-C., Brazier J.J., Yan M., Bargo P.R., Prahl S.A. (2004). Fluorescence-based optical sensor design for molecularly imprinted polymers. Sens. Actuators B Chem..

[107] Chen Y.-C., Wang Z., Yan M., Prahl S.A. (2006). Fluorescence anisotropy studies of molecularly imprinted polymers. Luminescence.

[108] Lieberzeit P.A., Halikias K., Afzal A., Dickert F.L. (2008). Polymers imprinted with PAH mixtures—comparing fluorescence and QCM sensors. Anal. Bioanal. Chem..

[109] Gehlen M.H. (2020). The centenary of the Stern-Volmer equation of fluorescence quenching: From the single line plot to the SV quenching map. J. Photochem. Photobiol. C Photochem. Rev..

[110] Salinas-Castillo A., Sánchez-Barragán I., Costa-Fernández J.M., Pereiro R., Ballesteros A., González J.M., Segura-Carretero A., Fernández-Gutiérrez A., Sanz-Medel A. (2005). Iodinated molecularly imprinted polymer for room temperature phosphorescence optosensing of fluoranthene. Chem. Commun..

[111] Fu Y., Finklea H.O. (2003). Quartz Crystal Microbalance Sensor for Organic Vapor Detection Based on Molecularly Imprinted Polymers. Anal. Chem..

[112] Bentekk/Dräger X-pid Gas Measurement Device Series. https://www.bentekk.com/.

[113] Step-Sensors Webseite! PID. https://www.step-sensor.de/english/environmental-measurement/pid/.

[114] Pacheco-Peña V., Beruete M., Rodríguez-Ulibarri P., Engheta N. (2019). On the performance of an ENZ-based sensor using transmission line theory and effective medium approach. New J. Phys..

[115] Reinecke T., Walter J.-G., Kobelt T., Ahrens A., Scheper T., Zimmermann S. (2018). Design and evaluation of split-ring resonators for aptamer-based biosensors. J. Sens. Sens. Syst..

[116] Diltemiz S.E., Hür D., Keçili R., Ersöz A., Say R. (2013). New synthesis method for 4-MAPBA monomer and using for the recognition of IgM and mannose with MIP-based QCM sensors. Analyst.

