# Supplementary material for: Molecularly Imprinted Polymer-Based Sensors for Priority Pollutants
Source: Sensors (Basel). 2021 Mar 31;21(7):2406. doi: 10.3390/s21072406 (PMC8037679; doi:10.3390/s21072406)
Supplement: Supplementary file 1 [file sensors-21-02406-s001.pdf]

Supplementary Materials

Molecularly Imprinted Polymer-Based Sensors for Priority Pollutants

Mashaalah Zarejousheghani [1,2,*], Parvaneh Rahimi [1], Helko Borsdorf [2], Stefan Zimmermann [3] and Yvonne Joseph [1]

1 Faculty of Materials Science and Materials Technology, Institute of Electronic and Sensor Materials, TU Bergakademie Freiberg, 09599 Freiberg, Germany; Parvaneh.Rahimi@esm.tu-freiberg.de (P.R.); Yvonne.Joseph@esm.tu-freiberg.de (Y.J.)
2 Department Monitoring and Exploration Technologies, UFZ-Helmholtz Centre for Environmental Research, 04318 Leipzig, Germany; helko.borsdorf@ufz.de
3 Department of Sensors and Measurement Technology, Institute of Electrical Engineering and Measurement Technology, Leibniz University Hannover, 30167 Hannover, Germany; zimmermann@geml.uni-hannover.de
* Correspondence: Mashaalah.Zarejousheghani@esm.tu-freiberg.de; Tel.: +49-373-139-3598

Table 1. Published manuscripts for the chemicals in EPA Priority Pollutant List.

Family group	Template/s	Target chemicals	Sensory concept	Sample	Ref.
BTEX	Toluene	Toluene	Chemiresistor	Gas	[1]
	1, 2, 3 Trimethoxybenzene	benzene, toluene and isomers of xylene	QCM	Gas	[2]
	phenol and acetoin	benzene and IMK	QCM	Gas	[3]
	Toluene	Toluene	Electrochemical	Gas	[4]
	Toluene	toluene, ethylbenzene and xylenes	Luminescence	Water	[5]
Nitrophenols	4-nitrophenol	4-nitrophenol	Electrochemical	Water	[6]
	4-nitrophenol	4-nitrophenol	Electrochemical	Water	[7]
	4-nitrophenol	4-nitrophenol	Luminescence	Water	[8]
	4-nitrophenol	4-nitrophenol	Electrochemical	Water	[9]
	4-nitrophenol	4-nitrophenol	Colorimetric	Water	[10]
	4-nitrophenol	4-nitrophenol	Luminescence	Water	[11]
	4-nitrophenol	4-nitrophenol	Luminescence	Water	[12]
	4-nitrophenol	4-nitrophenol	Luminescence	Water	[13]
	4-nitrophenol	4-nitrophenol	Luminescence	Water	[14]
	4-nitrophenol	4-nitrophenol	Colorimetric	Water	[15]
	4-nitrophenol	4-nitrophenol	Electrochemical	Water	[16]
	4-nitrophenol	4-nitrophenol	Luminescence	Water	[17]
	4-nitrophenol	4-nitrophenol	Electrochemical	Water	[18]
	4-nitrophenol	4-nitrophenol	Electrochemical	Water	[19]
	4-nitrophenol	4-nitrophenol	Luminescence	Water	[20]
	4-nitrophenol	4-nitrophenol	Luminescence	Water	[21]
	4-nitrophenol	4-nitrophenol	Luminescence	Water	[22]
	4-nitrophenol	4-nitrophenol	Luminescence	Water	[23]
	4-nitrophenol	4-nitrophenol	Electrochemical	Water	[24]
	4-nitrophenol	4-nitrophenol	Electrochemical	Water	[25]
	4-nitrophenol	4-nitrophenol	Electrochemical	Water	[26]
	2,4-dinitrophenol	2,4-dinitrophenol	Electrochemical	Water	[27]
	2,4-dinitrophenol	2,4-dinitrophenol	Electrochemical	Water	[28]

**Phenol & Chlorophenols**	Phenol	Phenol	QCM	Gas	[29]
	Phenol	Phenol	Colorimetric	Water	[30]
	Phenol	Phenol	Electrochemical	Water	[31]
	2,4,6-trichlorophenol	2,4,6-trichlorophenol	Luminescence	Water	[32]
	2,4,6-trichlorophenol	2,4,6-trichlorophenol	Luminescence	Water	[33]
	2,4-dichlorophenol	2,4-dichlorophenol	Luminescence	Water	[34]
	2,4-Dichlorophenol	2,4-dichlorophenol	Electrochemical	Water	[35]
	2,4-Dichlorophenol	2,4-Dichlorophenol	Electrochemical	Water	[36]
	2,4-Dichlorophenol	2,4-Dichlorophenol	Electrochemical	Water	[37]
**Nitrotoluenes & Nitrobenzene**	2,4-dinitrotoluene	2,4-dinitrotoluene	Luminescence	Water	[38]
	2,4-dinitrotoluene	2,4-dinitrotoluene	Luminescence	Gas	[39]
	2,4-dinitrotoluene	2,4-dinitrotoluene	Electrochemical/QCM	Water	[40]
	2,4-dinitrotoluene	2,4-dinitrotoluene	Luminescence	Water	[41]
	2,4-dinitrotoluene	2,4-dinitrotoluene	Colorimetric	Water	[42]
	2,4-dinitrotoluene	2,4-dinitrotoluene	Electrochemical	Water	[43]
	Nitrobenzene	Nitrobenzene	Chemiresistor	Gas	[44]
**Pesticides**	Dieldrin<br>Endosulfan<br>Hexachlorobenzene	Dieldrin<br>Endosulfan<br>Hexachlorobenzene	Colorimetric	Water	[45]
	Hexachlorobenzene	Hexachlorobenzene	QCM	Water	[46]
	Bisphenol A	DDT	Electrochemical	Water	[47]
	Endosulfan	Endosulfan	Electrochemical	Water	[48]
	Endosulfan	Endosulfan	QCM	Water	[49]
	Heptachlor	Heptachlor	Photoelectrochemical	Water	[50]
	Lindane	Lindane	Photoelectrochemical	Water	[51]
	Lindane	Lindane	Electrochemical	Water	[52]
**PAH**	Fluoranthene	Fluoranthene	Luminescence	Water	[53]
	Fluoranthene	Fluoranthene	Luminescence	Water	[54]
		Naphthalene	Luminescence	Gas	[55]
		benzo[a]pyrene	Luminescence	Water	[56]
		benzo[a]pyrene	Electrochemical	Water	[57]
		benzo[a]pyrene	Electrochemical	Water	[58]
		anthracene	Luminescence		[59]
		anthracene	Luminescence		[60]
		Naphthalene<br>anthracene	Chemiresistor	Water	[61]
	phenanthrene	phenanthrene	Luminescence	Milk	[62]
		pyrene	QCM<br>Luminescence		[63]
		1-hydroxypyrene<br>(is not in the list)	Electrochemical		[64]
**Phthalate esters**		diethylhexyl phthalate	Luminescence	Water	[65]
	dibutyl phthalate	dibutyl phthalate	Luminescence	Water	[66]
		Dibutyl phthalate	Luminescence	Water	[67]
		dibutyl phthalate	Luminescence	Water	[68]
		dibutylphthalate	Electrochemical		[69]

	Diisononyl Phthalate (is not in the list)	Electrochemical	[70]
Metal ions	Arsenic	Electrochemical	[71]
	Arsenic	Electrochemical	[72]
	Cadmium Copper	Electrochemical	[73]
	Cadmium	Luminescence	[74]
	Cadmium	Electrochemical	[75]
	Cadmium	Electrochemical	[76]
	Cadmium	Colorimetric	[77]
	Cadmium	Luminescence	[78]
	Chromium	Electrochemical	[79]
	Chromium	Luminescence	[80]
	Chromium	Luminescence	[81]
	Chromium	Electrochemical	[82]
	Copper Zinc	Electrochemical	[83]
	Copper	Electrochemical	[84]
	Copper	Electrochemical	[85]
	Copper	Electrochemical	[86]
	Cyanide	Electrochemical	[87]
	Lead	Electrochemical	[88]
	Lead	Electrochemical	[89]
	Lead	Electrochemical	[90]
	Lead (Conference paper)	QCM	[91]
	Lead	Electrochemical	[92]
	Lead	Electrochemical	[93]
	Mercury	Luminescence	[94]
	Mercury	Electrochemical	[95]
	Mercury	Electrochemical	[96]
	Mercury	Electrochemical	[97]
	Mercury	Electrochemical	[98]
	Mercury	Electrochemical	[99]
	Silver	Electrochemical	[100]
	Thallium	Electrochemical	[101]
	Zinc	Electrochemical	[102]

Reference

1. Alizadeh, T.; Rezaloo, F. Toluene chemiresistor sensor based on nano-porous toluene-imprinted polymer. *Int. J. Environ. Anal. Chem.* **2013**, *93*, 919–934, doi:10.1080/03067319.2012.708744.
2. Bhattacharyya Banerjee, M.; Pradhan, S.; Banerjee Roy, R.; Tudu, B.; Das, D.K.; Bandyopadhyay, R.; Pramanik, P. Detection of Benzene and Volatile Aromatic Compounds by Molecularly Imprinted Polymer-Coated Quartz Crystal Microbalance Sensor. *IEEE Sensors J.* **2019**, *19*, 885–892, doi:10.1109/JSEN.2018.2878926.
3. Hwang, M.J., Shim, W.G., Yoon, S.D.; Moon, H. Adsorption of toxic gases on molecularly imprinted polymer coated QCM: Measurements and modeling for partial pressure in gas mixture. *Adsorption* **2019**, *25*, 825–832, doi:10.1007/s10450-019-00074-w
4. Rongning Liang; Lusi Chen; Wei Qin. Potentiometric detection of chemical vapors using molecularly imprinted polymers as receptors. *Sci. Rep.* **2015**, *5*, 1–9, doi:10.1038/srep12462.

5. Sainz-Gonzalo, F.J.; Medina-Castillo, A.L.; Fernández-Sánchez, J.F.; Fernández-Gutiérrez, A. Synthesis and characterization of a molecularly imprinted polymer optosensor for TEXs-screening in drinking water. *Biosens. Bioelectron.* **2011**, *26*, 3331–3338, doi:10.1016/j.bios.2011.01.008.
6. Roy, A.C.; Nisha, V.S.; Dhand, C.; Ali, M.A.; Malhotra, B.D. Molecularly imprinted polyaniline-polyvinyl sulphonic acid composite based sensor for para-nitrophenol detection. *Anal. Chim. Acta* **2013**, *777*, 63–71, doi:10.1016/j.aca.2013.03.014.
7. Luo, J.; Cong, J.; Liu, J.; Gao, Y.; Liu, X. A facile approach for synthesizing molecularly imprinted graphene for ultrasensitive and selective electrochemical detecting 4-nitrophenol. *Anal. Chim. Acta* **2015**, *864*, 74–84, doi:10.1016/j.aca.2015.01.037.
8. Liu, J.; Chen, H.; Lin, Z.; Lin, J.-M. Preparation of Surface Imprinting Polymer Capped Mn-Doped ZnS Quantum Dots and Their Application for Chemiluminescence Detection of 4-Nitrophenol in Tap Water. *Analytical Chemistry* **2010**, *82*, 7380–7386, doi:10.1021/ac101510b.
9. Yan, K.; Yang, Y.; Zhu, Y.; Zhang, J. Highly Selective Self-Powered Sensing Platform for p-Nitrophenol Detection Constructed with a Photocathode-Based Photocatalytic Fuel Cell. *Analytical Chemistry* **2017**, *89*, 8599–8603, doi:10.1021/acs.analchem.7b02402.
10. Xue, F.; Meng, Z.; Wang, Y.; Huang, S.; Wang, Q.; Lu, W.; Xue, M. A molecularly imprinted colloidal array as a colorimetric sensor for label-free detection of p-nitrophenol. *Anal. Methods* **2014**, *6*, 831–837, doi:10.1039/C3AY42059K.
11. Jiang, L.; Liu, H.; Li, M.; Xing, Y.; Ren, X. Surface molecular imprinting on CdTe quantum dots for fluorescence sensing of 4-nitrophenol. *Anal. Methods* **2016**, *8*, 2226–2232, doi:10.1039/C5AY03160E.
12. Chang, L.; Chen, S.; Chu, J.; Li, X. Co-assembly of CdTe and Fe3O4 with molecularly imprinted polymer for recognition and separation of endocrine disrupting chemicals. *Appl. Surf. Sci.* **2013**, *284*, 745–749, doi:10.1016/j.apsusc.2013.08.004.
13. Li, W.; Zhang, H.; Chen, S.; Liu, Y.; Zhuang, J.; Lei, B. Synthesis of molecularly imprinted carbon dot grafted YVO4:Eu3+ for the ratiometric fluorescent determination of paranitrophenol. *Biosensors and Bioelectronics* **2016**, *86*, 706–713, doi:10.1016/j.bios.2016.07.034.
14. Zhou, Y.; Qu, Z.; Zeng, Y.; Zhou, T.; Shi, G. A novel composite of graphene quantum dots and molecularly imprinted polymer for fluorescent detection of paranitrophenol. *Biosensors and Bioelectronics* **2014**, *52*, 317–323, doi:10.1016/j.bios.2013.09.022.
15. XUE, F.; WANG, Y.-F.; WANG, Q.-H.; MENG, Z.-H.; XUE, M.; HUANG, S.-Y.; LU, W. Detection of p-Nitrophenol Using Molecularly Imprinted Colloidal Array. *Chinese Journal of Analytical Chemistry* **2012**, *40*, 218–223, doi:10.1016/S1872-2040(11)60532-X.
16. Zeng, Y.; Zhou, Y.; Zhou, T.; Shi, G. A novel composite of reduced graphene oxide and molecularly imprinted polymer for electrochemical sensing 4-nitrophenol. *Electrochimica Acta* **2014**, *130*, 504–511, doi:10.1016/j.electacta.2014.02.130.
17. Jialuo Yu; Xiaoyan Wang; Qi Kang; Jinhua Li; Dazhong Shen; Lingxin Chen. One-pot synthesis of a quantum dot-based molecular imprinting nanosensor for highly selective and sensitive fluorescence detection of 4-nitrophenol in environmental waters. *Environ. Sci.: Nano* **2017**, *4*, 493–502, doi:10.1039/C6EN00395H.
18. Saadati, F.; Ghahramani, F.; Shayani-jam, H.; Piri, F.; Yaftian, M.R. Synthesis and characterization of nanostructure molecularly imprinted polyaniline/graphene oxide composite as highly selective electrochemical sensor for detection of p-nitrophenol. *Journal of the Taiwan Institute of Chemical Engineers* **2018**, *86*, 213–221, doi:10.1016/j.jtice.2018.02.019.
19. Guilin Xu; Lingling Yang; Min Zhong; Chen Li; Xiaojing Lu; Xianwen Kan. Selective recognition and electrochemical detection of p -nitrophenol based on a macroporous imprinted polymer containing gold nanoparticles. *Microchim Acta* **2013**, *180*, 1461–1469, doi:10.1007/s00604-013-1090-8.
20. Tongfan Hao; Xiao Wei; Yijing Nie; Yeqing Xu; Yongsheng Yan; Zhiping Zhou. An eco-friendly molecularly imprinted fluorescence composite material based on carbon dots for fluorescent detection of 4-nitrophenol. *Microchim Acta* **2016**, *183*, 2197–2203, doi:10.1007/s00604-016-1851-2.
21. Mingyue Liu; Zhao Gao; Yanjun Yu; Rongxin Su; Renliang Huang; Wei Qi; Zhimin He. Molecularly Imprinted Core-Shell CdSe@SiO 2 /CDs as a Ratiometric Fluorescent Probe for 4-Nitrophenol Sensing. *Nanoscale Res Lett* **2018**, *13*, 1–9, doi:10.1186/s11671-018-2440-6.
22. Yingchun Wang; Ningwei Wang; Xiaoni Ni; Qianqian Jiang; Wenming Yang; Weihong Huang; Wanzhen Xu. A core–shell CdTe quantum dots molecularly imprinted polymer for recognizing and detecting p-nitrophenol based on computer simulation. *RSC Adv.* **2015**, *5*, 73424–73433, doi:10.1039/C5RA06889D.
23. Xiao Wei; Zhiping Zhou; Tongfan Hao; Hongji Li; Yanzhuo Zhu; Lin Gao; Yongsheng Yan. A novel molecularly imprinted polymer thin film at surface of ZnO nanorods for selective fluorescence detection of para-nitrophenol. *RSC Adv.* **2015**, *5*, 44088–44095, doi:10.1039/C5RA05093F.
24. Guo, X.; Zhou, H.; Fan, T.; Di Zhang. Electrochemical detection of p-nitrophenol on surface imprinted gold with lamellar-ridge architecture. *Sensors and Actuators B: Chemical* **2015**, *220*, 33–39, doi:10.1016/j.snb.2015.05.042.

25. Alizadeh, T.; Ganjali, M.R.; Norouzi, P.; Zare, M.; Zeraatkar, A. A novel high selective and sensitive para-nitrophenol voltammetric sensor, based on a molecularly imprinted polymer-carbon paste electrode. *Talanta* **2009**, *79*, 1197–1203, doi:10.1016/j.talanta.2009.02.051.
26. Hu, Y.; Zhang, Z.; Zhang, H.; Luo, L.; Yao, S. Sensitive and selective imprinted electrochemical sensor for p-nitrophenol based on ZnO nanoparticles/carbon nanotubes doped chitosan film. *Thin Solid Films* **2012**, *520*, 5314–5321, doi:10.1016/j.tsf.2011.11.083.
27. Jing, T.; Xia, H.; Niu, J.; Zhou, Y.; Dai, Q.; Hao, Q.; Zhou, Y.; Mei, S. Determination of trace 2,4-dinitrophenol in surface water samples based on hydrophilic molecularly imprinted polymers/nickel fiber electrode. *Biosens. Bioelectron.* **2011**, *26*, 4450–4456, doi:10.1016/j.bios.2011.05.001.
28. Liu, Y.; Zhu, L.; Zhang, Y.; Tang, H. Electrochemical sensoring of 2,4-dinitrophenol by using composites of graphene oxide with surface molecular imprinted polymer. *Sensors and Actuators B: Chemical* **2012**, *171-172*, 1151–1158, doi:10.1016/j.snb.2012.06.054.
29. Fu, Y.; Finklea, H.O. Quartz Crystal Microbalance Sensor for Organic Vapor Detection Based on Molecularly Imprinted Polymers. *Analytical Chemistry* **2003**, *75*, 5387–5393, doi:10.1021/ac034523b.
30. Sergeyeva, T.A.; Chelyadina, D.S.; Gorbach, L.A.; Brovko, O.O.; Piletska, E.V.; Piletsky, S.A.; Sergeeva, L.M.; El'skaya, A.V. Colorimetric biomimetic sensor systems based on molecularly imprinted polymer membranes for highly-selective detection of phenol in environmental samples. *Biopolym. Cell* **2014**, *30*, 209–215, doi:10.7124/bc.000898.
31. S. Rahmadhani; H. Setiyanto; M. A. Zulfikar. Electropolymerized of aniline as a new molecularly imprinted polymer for determination of phenol: A study for phenol sensor. In *2017 International Seminar on Sensors, Instrumentation, Measurement and Metrology (ISSIMM).* 2017 International Seminar on Sensors, Instrumentation, Measurement and Metrology (ISSIMM), 2017; pp 124–128.
32. Lin, X.; Wu, Y.; Hao, Y.; Sun, Q.; Yan, Y.; Li, C. Sensitive and Selective Determination of 2,4,6-Trichlorophenol Using a Molecularly Imprinted Polymer Based on Zinc Oxide Quantum Dots. *Analytical Letters* **2018**, *51*, 1578–1591, doi:10.1080/00032719.2017.1384480.
33. Xiao Wei; Miaomiao Yu; Chen Li; Xinghui Gong; Fang Qin; Zhenhong Wang. Magnetic nanoparticles coated with a molecularly imprinted polymer doped with manganese-doped ZnS quantum dots for the determination of 2,4,6-trichlorophenol. *Microchim Acta* **2018**, *185*, 1–6, doi:10.1007/s00604-018-2742-5.
34. Wei, X.; Zhou, Z.; Hao, T.; Li, H.; Xu, Y.; Lu, K.; Wu, Y.; Dai, J.; Pan, J.; Yan, Y. Highly-controllable imprinted polymer nanoshell at the surface of silica nanoparticles based room-temperature phosphorescence probe for detection of 2,4-dichlorophenol. *Anal. Chim. Acta* **2015**, *870*, 83–91, doi:10.1016/j.aca.2015.02.025.
35. Liu, B.; Cang, H.; Jin, J. Molecularly Imprinted Polymers Based Electrochemical Sensor for 2,4-Dichlorophenol Determination. *Polymers (Basel)* **2016**, *8*, doi:10.3390/polym8080309.
36. Liang, Y.; Yu, L.; Yang, R.; Li, X.; Qu, L.; Li, J. High sensitive and selective graphene oxide/molecularly imprinted polymer electrochemical sensor for 2,4-dichlorophenol in water. *Sensors and Actuators B: Chemical* **2017**, *240*, 1330–1335, doi:10.1016/j.snb.2016.08.137.
37. Liu, Y.; Liang, Y.; Yang, R.; Li, J.; Qu, L. A highly sensitive and selective electrochemical sensor based on polydopamine functionalized graphene and molecularly imprinted polymer for the 2,4-dichlorophenol recognition and detection. *Talanta* **2019**, *195*, 691–698, doi:10.1016/j.talanta.2018.11.052.
38. Stringer, R.C.; Gangopadhyay, S.; Grant, S.A. Comparison of molecular imprinted particles prepared using precipitation polymerization in water and chloroform for fluorescent detection of nitroaromatics. *Anal. Chim. Acta* **2011**, *703*, 239–244, doi:10.1016/j.aca.2011.07.034.
39. Qingyuan Niu; Kezheng Gao; Zhihui Lin; Wenhui Wu. Surface molecular-imprinting engineering of novel cellulose nanofibril/conjugated polymer film sensors towards highly selective recognition and responsiveness of nitroaromatic vapors. *Chem. Commun.* **2013**, *49*, 9137–9139, doi:10.1039/C3CC44705G.
40. Huynh, T.-P.; Sosnowska, M.; Sobczak, J.W.; Kc, C.B.; Nesterov, V.N.; D'Souza, F.; Kutner, W. Simultaneous chronoamperometry and piezoelectric microgravimetry determination of nitroaromatic explosives using molecularly imprinted thiophene polymers. *Analytical Chemistry* **2013**, *85*, 8361–8368, doi:10.1021/ac4017677.
41. Stringer, R.C.; Gangopadhyay, S.; Grant, S.A. Detection of nitroaromatic explosives using a fluorescent-labeled imprinted polymer. *Analytical Chemistry* **2010**, *82*, 4015–4019, doi:10.1021/ac902838c.
42. LU, W.; Dong, X.; Qiu, L.; Yan, Z.; Meng, Z.; XUE, M.; He, X.; Liu, X. Colorimetric sensor arrays based on pattern recognition for the detection of nitroaromatic molecules. *J. Hazard. Mater.* **2017**, *326*, 130–137, doi:10.1016/j.jhazmat.2016.12.024.
43. Nie, D.; Han, Z.; Yu, Y.; Shi, G. Composites of multiwalled carbon nanotubes/polyethyleneimine (MWCNTs/PEI) and molecularly imprinted polymers for dinitrotoluene recognition. *Sensors and Actuators B: Chemical* **2016**, *224*, 584–591, doi:10.1016/j.snb.2015.10.103.

44. Alizadeh, T.; Hamedsoltani, L. Graphene/graphite/molecularly imprinted polymer nanocomposite as the highly selective gas sensor for nitrobenzene vapor recognition. *Journal of Environmental Chemical Engineering* **2014**, *2*, 1514–1526, doi:10.1016/j.jece.2014.07.007.
45. Silverio, O.V.; So, R.C.; Elnar, K.J.S.; Malapit, C.A.; Nepomuceno, M.C.M. Development of dieldrin, endosulfan, and hexachlorobenzene-imprinted polymers for dye-displacement array sensing. *J. Appl. Polym. Sci.* **2017**, *134*, doi:10.1002/app.44401.
46. Das, K.; Penelle, J.; Rotello, V.M. Selective Picomolar Detection of Hexachlorobenzene in Water Using a Quartz Crystal Microbalance Coated with a Molecularly Imprinted Polymer Thin Film. *Langmuir* **2003**, *19*, 3921–3925, doi:10.1021/la026781u.
47. Miao, J.; Liu, A.; Wu, L.; Yu, M.; Wei, W.; Liu, S. Magnetic ferroferric oxide and polydopamine molecularly imprinted polymer nanocomposites based electrochemical impedance sensor for the selective separation and sensitive determination of dichlorodiphenyltrichloroethane (DDT). *Anal. Chim. Acta* **2020**, *1095*, 82–92, doi:10.1016/j.aca.2019.10.027.
48. Yohandri Bow; Edy Sutriyono; Subriyer Nasir; Iskhaq Iskandar. Molecularly Imprinted Polymers (MIP) Based Electrochemical Sensor for Detection of Endosulfan Pesticide **2017**.
49. Nan Liu; Jianguang Han; Zhen Liu; Lijie Qu; Zhixian Gao. Rapid detection of endosulfan by a molecularly imprinted polymer microsphere modified quartz crystal microbalance. *Anal. Methods* **2013**, *5*, 4442–4447, doi:10.1039/C3AY40697K.
50. Panpan Wang; Guoqiang Sun; Lei Ge; Shenguang Ge; Jinghua Yu; Mei Yan. Photoelectrochemical lab-on-paper device based on molecularly imprinted polymer and porous Au-paper electrode. *Analyst* **2013**, *138*, 4802–4811, doi:10.1039/C3AN00694H.
51. Panpan Wang; Lei Ge; Meng Li; Weiping Li; Long Li; Yanhu Wang; Jinghua Yu. Photoelectrochemical Sensor Based on Molecularly Imprinted Polymer-Coated TiO 2 Nanotubes for Lindane Specific Recognition and Detection. *J Inorg Organomet Polym* **2013**, *23*, 703–711, doi:10.1007/s10904-013-9836-7.
52. Anirudhan, T.S.; Alexander, S. Design and fabrication of molecularly imprinted polymer-based potentiometric sensor from the surface modified multiwalled carbon nanotube for the determination of lindane (γ-hexachlorocyclohexane), an organochlorine pesticide. *Biosensors and Bioelectronics* **2015**, *64*, 586–593, doi:10.1016/j.bios.2014.09.074.
53. Sánchez-Barragán, I.; Costa-Fernández, J.M.; Pereiro, R.; Sanz-Medel, A.; Salinas, A.; Segura, A.; Fernández-Gutiérrez, A.; Ballesteros, A.; González, J.M. Molecularly Imprinted Polymers Based on Iodinated Monomers for Selective Room-Temperature Phosphorescence Optosensing of Fluoranthene in Water. *Analytical Chemistry* **2005**, *77*, 7005–7011, doi:10.1021/ac050400a.
54. A. Salinas-Castillo; I. Sánchez-Barragán; J. M. Costa-Fernández; R. Pereiro; A. Ballesteros; J. M. González; A. Segura-Carretero; A. Fernández-Gutiérrez; A. Sanz-Medel. Iodinated molecularly imprinted polymer for room temperature phosphorescence optosensing of fluoranthene. *Chem. Commun.* **2005**, 3224–3226, doi:10.1039/B502706C.
55. A. V. Koshkin; V. A. Sazhnikov; A. Yu. Men'shikova; G. A. Pankova; T. G. Evseeva; M. V. Alfimov. Naphthalene vapor sorption by polymer nanoparticles with molecularly imprinted shells. *Nanotechnol Russia* **2012**, *7*, 15–21, doi:10.1134/S1995078012010120.
56. Traviesa-Alvarez, J.M.; Sánchez-Barragán, I.; Costa-Fernández, J.M.; Pereiro, R.; Sanz-Medel, A. Room temperature phosphorescence optosensing of benzoapyrene in water using halogenated molecularly imprinted polymers. *Analyst* **2007**, *132*, 218–223, doi:10.1039/b616919h.
57. Udomsap, D.; Branger, C.; Culioli, G.; Dollet, P.; Brisset, H. A versatile electrochemical sensing receptor based on a molecularly imprinted polymer. *Chem. Commun. (Camb)* **2014**, *50*, 7488–7491, doi:10.1039/c4cc02658f.
58. Udomsap, D.; Brisset, H.; Culioli, G.; Dollet, P.; Laatikainen, K.; Siren, H.; Branger, C. Electrochemical molecularly imprinted polymers as material for pollutant detection. *Materials Today Communications* **2018**, *17*, 458–465, doi:10.1016/j.mtcomm.2018.10.019.
59. Chen, Y.-C.; Wang, Z.; Yan, M.; Prahl, S.A. Fluorescence anisotropy studies of molecularly imprinted polymers. *Luminescence* **2006**, *21*, 7–14, doi:10.1002/bio.874.
60. Chen, Y.-C.; Brazier, J.J.; Yan, M.; Bargo, P.R.; Prahl, S.A. Fluorescence-based optical sensor design for molecularly imprinted polymers. *Sensors and Actuators B: Chemical* **2004**, *102*, 107–116, doi:10.1016/j.snb.2004.02.044.
61. Latif, U.; Ping, L.; Dickert, F.L. Conductometric Sensor for PAH Detection with Molecularly Imprinted Polymer as Recognition Layer. *Sensors (Basel)* **2018**, *18*, doi:10.3390/s18030767.
62. Li, H.; Wang, L. Highly Selective Detection of Polycyclic Aromatic Hydrocarbons Using Multifunctional Magnetic–Luminescent Molecularly Imprinted Polymers. *ACS Applied Materials & Interfaces* **2013**, *5*, 10502–10509, doi:10.1021/am4020605.
63. Peter A. Lieberzeit; Konstantin Halikias; Adeel Afzal; Franz L. Dickert. Polymers imprinted with PAH mixtures—comparing fluorescence and QCM sensors. *Anal Bioanal Chem* **2008**, *392*, 1405–1410, doi:10.1007/s00216-008-2413-1.
64. Nicole Kirsch; John P. Hart; David J. Bird; Richard W. Luxton; David V. McCalley. Towards the development of molecularly imprinted polymer based screen-printed sensors for metabolites of PAHs. *Analyst* **2001**, *126*, 1936–1941, doi:10.1039/B108008N.

65. Wang, Y.; Zhou, Z.; Xu, W.; Luan, Y.; Lu, Y.; Yang, Y.; Liu, T.; Li, S.; Yang, W. Surface molecularly imprinted polymers based ZnO quantum dots as fluorescence sensors for detection of diethylhexyl phthalate with high sensitivity and selectivity. *Polym. Int.* **2018**, *67*, 1003–1010, doi:10.1002/pi.5596.
66. Li, T.; Gao, Z.; Wang, N.; Zhou, Z.; Xu, W.; Zheng, J.; Yang, W. Synthesis and evaluation of a molecularly imprinted polymer with high-efficiency recognition for dibutyl phthalate based on Mn-doped ZnS quantum dots. *RSC Adv.* **2016**, *6*, 54615–54622, doi:10.1039/C6RA04663K.
67. Xu, W.; Li, T.; Huang, W.; Luan, Y.; Yang, Y.; Li, S.; Yang, W. A magnetic fluorescence molecularly imprinted polymer sensor with selectivity for dibutyl phthalate via Mn doped ZnS quantum dots. *RSC Adv.* **2017**, *7*, 51632–51639, doi:10.1039/C7RA09145A.
68. Zhou, Z.; Li, T.; Xu, W.; Huang, W.; Wang, N.; Yang, W. Synthesis and characterization of fluorescence molecularly imprinted polymers as sensor for highly sensitive detection of dibutyl phthalate from tap water samples. *Sensors and Actuators B: Chemical* **2017**, *240*, 1114–1122, doi:10.1016/j.snb.2016.09.092.
69. Li, X.; Wang, X.; Li, L.; Duan, H.; Luo, C. Electrochemical sensor based on magnetic graphene oxide@gold nanoparticles-molecular imprinted polymers for determination of dibutyl phthalate. *Talanta* **2015**, *131*, 354–360, doi:10.1016/j.talanta.2014.07.028.
70. X. Zhao; X. Ju; S. Qiu; W. Hu; L. Yang; J. Zhang. Fast and Sensitive Detection of Diisononyl Phthalate in Liquor Sample by Molecularly Imprinted Polymer Based Electrochemical Sensor. *Russ J Electrochem* **2018**, *54*, 636–643, doi:10.1134/S1023193518080074.
71. Alizadeh, T.; Rashedi, M. Synthesis of nano-sized arsenic-imprinted polymer and its use as As3+ selective ionophore in a potentiometric membrane electrode: Part 1. *Anal. Chim. Acta* **2014**, *843*, 7–17, doi:10.1016/j.aca.2014.06.052.
72. Alizadeh, T.; Rashedi, M.; Hanifehpour, Y.; Joo, S.W. Improvement of durability and analytical characteristics of arsenic-imprinted polymer-based PVC membrane electrode via surface modification of nano-sized imprinted polymer particles: part 2. *Electrochimica Acta* **2015**, *178*, 877–885, doi:10.1016/j.electacta.2015.08.045.
73. Bali Prasad, B.; Jauhari, D.; Verma, A. A dual-ion imprinted polymer embedded in sol–gel matrix for the ultra trace simultaneous analysis of cadmium and copper. *Talanta* **2014**, *120*, 398–407, doi:10.1016/j.talanta.2013.12.036.
74. Alizadeh, T.; Sharifi, A.R.; Ganjali, M.R. A new bio-compatible Cd 2+ -selective nanostructured fluorescent imprinted polymer for cadmium ion sensing in aqueous media and its application in bio imaging in Vero cells. *RSC Adv.* **2020**, *10*, 4110–4117, doi:10.1039/C9RA06910K.
75. Alizadeh, T.; Ganjali, M.R.; Nourozi, P.; Zare, M.; Hoseini, M. A carbon paste electrode impregnated with Cd2+ imprinted polymer as a new and high selective electrochemical sensor for determination of ultra-trace Cd2+ in water samples. *Journal of Electroanalytical Chemistry* **2011**, *657*, 98–106, doi:10.1016/j.jelechem.2011.03.029.
76. Dahaghin, Z.; Kilmartin, P.A.; Mousavi, H.Z. Determination of cadmium(II) using a glassy carbon electrode modified with a Cd-ion imprinted polymer. *Journal of Electroanalytical Chemistry* **2018**, *810*, 185–190, doi:10.1016/j.jelechem.2018.01.014.
77. Huang, K.; Chen, Y.; Zhou, F.; Zhao, X.; Liu, J.; Mei, S.; Zhou, Y.; Jing, T. Integrated ion imprinted polymers-paper composites for selective and sensitive detection of Cd(II) ions. *J. Hazard. Mater.* **2017**, *333*, 137–143, doi:10.1016/j.jhazmat.2017.03.035.
78. Shuhuai Li; Xionghui Ma; Chaohai Pang; Hai Tian; Zhi Xu; Yan Yang; Daizhu Lv; Huilin Ge. Fluorometric aptasensor for cadmium(II) by using an aptamer-imprinted polymer as the recognition element. *Microchim Acta* **2019**, *186*, 1–7, doi:10.1007/s00604-019-3886-7.
79. Alizadeh, T.; Mirzaee, S.; Rafiei, F. All-solid-state Cr(III)-selective potentiometric sensor based on Cr(III)-imprinted polymer nanomaterial/MWCNTs/carbon nanocomposite electrode. *International Journal of Environmental Analytical Chemistry* **2017**, *97*, 1283–1297, doi:10.1080/03067319.2017.1408804.
80. Lu, H.; Xu, S. Dual channel ion imprinted fluorescent polymers for dual mode simultaneous chromium speciation analysis. *Analyst* **2020**, *145*, 2661–2668, doi:10.1039/d0an00098a.
81. Meng Yuan Zhang; Ren Feng Huang; Xiao Guo Ma; Li Hui Guo; Ying Wang; Yin Ming Fan. Selective fluorescence sensor based on ion-imprinted polymer-modified quantum dots for trace detection of Cr(VI) in aqueous solution. *Anal Bioanal Chem* **2019**, *411*, 7165–7175, doi:10.1007/s00216-019-02100-w.
82. Alizadeh, T.; Rafiei, F.; Hamidi, N.; Ganjali, M.R. A new electrochemical sensing platform for Cr(III) determination based on nano-structured Cr(III)-imprinted polymer-modified carbon composite electrode. *Electrochimica Acta* **2017**, *247*, 812–819, doi:10.1016/j.electacta.2017.07.081.
83. Kumar, D.; Madhuri, R.; Prasad Tiwari, M.; Sinha, P.; Bali Prasad, B. Molecularly Imprinted Polymer-modified Electrochemical Sensor For Simultaneous Determination Of Copper And Zinc. *AML* **2011**, *2*, 294–297, doi:10.5185/amlett.indias.207.
84. Zhihua, W.; Xiaole, L.; Jianming, Y.; Yaxin, Q.; Xiaoquan, L. Copper(II) determination by using carbon paste electrode modified with molecularly imprinted polymer. *Electrochimica Acta* **2011**, *58*, 750–756, doi:10.1016/j.electacta.2011.10.034.

85. Topcu, C.; Lacin, G.; Yilmaz, V.; Coldur, F.; Caglar, B.; Cubuk, O.; Isildak, I. Electrochemical Determination of Copper(II) in Water Samples Using a Novel Ion-Selective Electrode Based on a Graphite Oxide–Imprinted Polymer Composite. *Analytical Letters* **2018**, *51*, 1890–1910, doi:10.1080/00032719.2017.1395035.
86. Hamid Reza Rajabi; Arezoo Zarezadeh; Gholamreza Karimipour. Porphyrin based nano-sized imprinted polymer as an efficient modifier for the design of a potentiometric copper carbon paste electrode. *RSC Adv.* **2017**, *7*, 14923–14931, doi:10.1039/C6RA27288F.
87. Alizadeh, T.; Sabzi, R.E.; Alizadeh, H. Synthesis of nano-sized cyanide ion-imprinted polymer via non-covalent approach and its use for the fabrication of a CN(-)-selective carbon nanotube impregnated carbon paste electrode. *Talanta* **2016**, *147*, 90–97, doi:10.1016/j.talanta.2015.09.043.
88. Hu, S.; Xiong, X.; Huang, S.; Lai, X. Preparation of Pb(II) Ion Imprinted Polymer and Its Application as the Interface of an Electrochemical Sensor for Trace Lead Determination. *Anal. Sci.* **2016**, *32*, 975–980, doi:10.2116/analsci.32.975.
89. Bojdi, M.K.; Mashhadizadeh, M.H.; Behbahani, M.; Farahani, A.; Davarani, S.S.H.; Bagheri, A. Synthesis, characterization and application of novel lead imprinted polymer nanoparticles as a high selective electrochemical sensor for ultra-trace determination of lead ions in complex matrixes. *Electrochimica Acta* **2014**, *136*, 59–65, doi:10.1016/j.electacta.2014.05.095.
90. Dahaghin, Z.; Kilmartin, P.A.; Mousavi, H.Z. Novel ion imprinted polymer electrochemical sensor for the selective detection of lead(II). *Food Chem.* **2020**, *303*, 125374, doi:10.1016/j.foodchem.2019.125374.
91. Achour, B.; Mazouz, Z.; Fourati, N.; Zerrouki, C.; Aloui, N.; Yaakoubi, N.; Othmane, A.; Kalfat, R. Ultrasensitive Ion Imprinted Polypyrole Polymer Based Piezoelectric Sensors for Selective Detection of Lead Ions. In *IEEE SENSORS 2018*, 2018 conference proceedings : October 28-31, 2018, Pullman Aerocity, New Delhi, India. 2018 IEEE Sensors, New Delhi, 10/28/2018 - 10/31/2018; IEEE: Piscataway, NJ, 2018; pp 1–4, ISBN 978-1-5386-4707-3.
92. Alizadeh, T.; Amjadi, S. Preparation of nano-sized Pb2+ imprinted polymer and its application as the chemical interface of an electrochemical sensor for toxic lead determination in different real samples. *J. Hazard. Mater.* **2011**, *190*, 451–459, doi:10.1016/j.jhazmat.2011.03.067.
93. Luo, X.; Huang, W.; Shi, Q.; Xu, W.; Luan, Y.; Yang, Y.; Wang, H.; Yang, W. Electrochemical sensor based on lead ion-imprinted polymer particles for ultra-trace determination of lead ions in different real samples. *RSC Adv.* **2017**, *7*, 16033–16040, doi:10.1039/C6RA25791G.
94. Güney, O.; Cebeci, F.Ç. Molecularly imprinted fluorescent polymers as chemosensors for the detection of mercury ions in aqueous media. *J. Appl. Polym. Sci.* **2010**, *117*, 2373–2379, doi:10.1002/app.32077.
95. Shirzadmehr, A.; Afkhami, A.; Madrakian, T. A new nano-composite potentiometric sensor containing an Hg2+-ion imprinted polymer for the trace determination of mercury ions in different matrices. *Journal of Molecular Liquids* **2015**, *204*, 227–235, doi:10.1016/j.molliq.2015.01.014.
96. Ghanei-Motlagh, M.; Taher, M.A.; Heydari, A.; Ghanei-Motlagh, R.; Gupta, V.K. A novel voltammetric sensor for sensitive detection of mercury(II) ions using glassy carbon electrode modified with graphene-based ion imprinted polymer. *Mater. Sci. Eng. C Mater. Biol. Appl.* **2016**, *63*, 367–375, doi:10.1016/j.msec.2016.03.005.
97. Velempini, T.; Pillay, K.; Mbianda, X.Y.; Arotiba, O.A. Application of a Polypyrrole/Carboxy Methyl Cellulose Ion Imprinted Polymer in the Electrochemical Detection of Mercury in Water. *Electroanalysis* **2018**, *30*, 2612–2619, doi:10.1002/elan.201800445.
98. Alizadeh, T.; Ganjali, M.R.; Zare, M. Application of an Hg2+ selective imprinted polymer as a new modifying agent for the preparation of a novel highly selective and sensitive electrochemical sensor for the determination of ultratrace mercury ions. *Anal. Chim. Acta* **2011**, *689*, 52–59, doi:10.1016/j.aca.2011.01.036.
99. Taher Alizadeh; Negin Hamidi; Mohamad Reza Ganjali; Faride Rafiei. Determination of subnanomolar levels of mercury (II) by using a graphite paste electrode modified with MWCNTs and Hg(II)-imprinted polymer nanoparticles. *Microchim Acta* **2018**, *185*, 1–9, doi:10.1007/s00604-017-2534-3.
100. Zhiani, R.; Ghanei-Motlag, M.; Razavipanah, I. Selective voltammetric sensor for nanomolar detection of silver ions using carbon paste electrode modified with novel nanosized Ag(I)-imprinted polymer. *Journal of Molecular Liquids* **2016**, *219*, 554–560, doi:10.1016/j.molliq.2016.03.052.
101. Nasiri-Majd, M.; Taher, M.A.; Fazelirad, H. Synthesis and application of nano-sized ionic imprinted polymer for the selective voltammetric determination of thallium. *Talanta* **2015**, *144*, 204–209, doi:10.1016/j.talanta.2015.05.058.
102. Behnia, N.; Asgari, M.; Feizbakhsh, A. Sub-nanomolar detection of zinc on the ion-imprinted polymer modified glassy carbon electrode. *Journal of Environmental Chemical Engineering* **2015**, *3*, 271–276, doi:10.1016/j.jece.2014.11.008.
